# Themes of Biological Inheritance in Early Nineteenth Century Sheep Breeding as Revealed by J. M. Ehrenfels

**DOI:** 10.3390/genes13081311

**Published:** 2022-07-23

**Authors:** Péter Poczai, Jorge A. Santiago-Blay

**Affiliations:** 1 Finnish Museum of Natural History, University of Helsinki, P.O. Box 7, FI-00014 Helsinki, Finland; 2 Museomics Research Group, Viikki Plant Science Centre (ViPS), University of Helsinki, P.O. Box 65, FI-00014 Helsinki, Finland; 3 Institute of Advanced Studies Kőszeg (iASK), P.O. Box 4, H-9731 Kőszeg, Hungary; 4 Department of Paleobiology, National Museum of Natural History, Washington, DC 20560, USA; 5 Science, The Pennsylvania State University, 1031 Edgecomb Avenue, York, PA 17403, USA

**Keywords:** deviations, genetic force, heredity, inbreeding, sheep’s wool

## Abstract

Among the so-called sheep breeders interested in biological inheritance in the late eighteenth and early nineteenth centuries and well before Gregor Johann Mendel, J. M. Ehrenfels (1767–1843) produced some of the most cogent writings on the subject. Although earlier in his career Ehrenfels was a strong advocate of environmental factors as influencers on the appearance of organisms, as a result of his discussions with Imre Festetics, he became convinced that whatever is passed from parents to progeny is more important and it is dependent on a “genetic force, the mother of all living things”. The sheep breeders kept issues of inheritance at the forefront of the Central European cultural context late into the nineteenth century.

## 1. Introduction

*How could varieties be formed into consolidated, constant sheep breeds? This question is difficult to answer depending on whether we believe in nature; and not yet a mathematically solvable problem*

[1] (p. 129).

For more than a century, the textbook teaching of genetics emphasized Gregor Johann Mendel (1822–1884) and his experiments in the hybridization of peas (*Pisum sativum* L.) [2], which constitute a foundation of genetics at the organismic level. Mendel’s pea work was connected to cytology (observations on chromosomes made by Nettie Stevens circa 1905 as the cytological bases of inheritance [3]), and later the advancements in molecular biology beginning in the 1930s. Nevertheless, the textbook rendition of Mendel’s work is not only largely devoid of the historical context in which his work took place, including professors and colleagues [4] as well as, possibly, Charles Darwin (1809–1882) [5,6], but also ignores the cultural and political context in which Mendel was immersed and what other Central European authors were writing in the first half of the nineteenth century [7] (pp. 275–277), [8] (pp. 24–81).

One such group of scholars were the so-called sheep breeders (1806–1898) of the Moravian Agricultural and Natural Science Society (MAS, also known as the *Ackerbaugesellschaft*), who were generally well-to-do individuals living in the Habsburg Empire (nowadays Central Europe). In addition to their interest in breeding sheep for profit, especially when the Empire was at war against Napoleonic France (1803–1815), the sheep breeders sought to address the practical issue of consistently generating sheep with high-quality traits desirable to humans (e.g., wool, meat, and milk). In doing so, some of the breeders were addressing a great mystery of nature: how does heredity happen?

**Figure 1:**
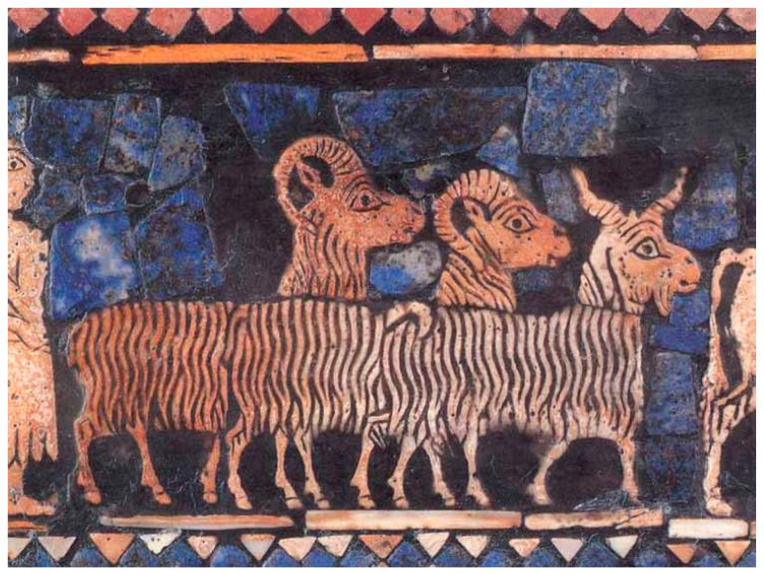
The Standard of Ur, a Sumerian artifact from the Early Dynastic III period c. 2500 bce with mosaic scenes made from shell, red limestone, and lapis lazuli depicting ancient sheep and goat breeds. Photo courtesy of the British Museum (Item No. 12561001).

In the Neolithic period, the domestication of plants and animals was an important turning point in human progress [9]. Evolutionary scientists, including Darwin, have long been fascinated by this process because it provided a succession of separate long-term hereditary experiments in which plants and animals were chosen for specific qualities [10]. Breeders, who were eyewitnesses to the processes for millennia, gathered fundamental evidence of how traits pass down from one generation to another [11]. Sheep, with their distinctive coat, milk, and meat, were particularly sought after among domesticated animals. The domestication of this mammal dates back as far as 11,000–9000 bce in Mesopotamia with prominent ovine symbols in various civilizations [12] (Figure 1).

The birth of basic concepts about biological inheritance have rendered the pre-Mendelian time and location, early nineteenth-century Central Europe, essential to the study of the intersections between natural knowledge and scientific breeding. In particular, a sizable and still-growing number of historians have demonstrated how new concepts of heredity that is, of physical resemblance transferred from parent to progeny through time, evolved in part from new markets in “blood” that formed during the eighteenth and nineteenth centuries [13] (pp. 47–48). Sheep breeds sold and valued for their ability to pass on their good qualities to their offspring served as highly publicized models of transmissible bodily change, models that would have far-reaching effects on racial, evolutionary, and ultimately eugenic and genetic theory [14,15,16,17,18]. Simultaneously, the little-known textbooks, body standards, principles of breeding, and actual animals produced during this period continue to strongly influence the millions of animals whose lives comprise modern meat, dairy, and wool production systems. As the commodification of “blood” in the eighteenth and nineteenth centuries is an obvious precursor to the rapid commodification of genetic material at the end of the twentieth century, the study of “biocapital” in the history of the life sciences has amplified the interest in selective breeding [13,19,20,21].

Pre-Mendelian scholars have demonstrated how unseen aspects of heredity became a new type of commons, swiftly privatized, and subject to speculation, with tremendous scientific, social, and environmental repercussions [8] (pp. 69–72). It is, however, largely concerned with the time of commodification, as is the case with a significant deal of scientific history focused on heredity. To understand the evolving forms of knowledge that shaped the processes of animal and crop production, we must examine a different scale of life, not the hidden capabilities of blood or DNA, but living animals and plants, the fields or pastures where they lived, and the social institutions and forms of knowledge that created the fields [22,23]. Such knowledge includes a considerably broader range of topics, including what we could today term studies of animal physiology, genetics, selection, and evolution.

Pursuant to the examination of little-known published manuscripts by authors associated with the sheep breeders well before Mendel and printed in Fraktur German, we are beginning to uncover the contents of their writings and how they are connected to Mendel’s later work. In our work we examine the papers written by Josef Michael Freiherr von Ehrenfels (1767–1843) and re-publish the transcription and English translation of his selected works concerning biological inheritance (see Supplementary Material S1). In this paper, we briefly describe what we think are the principles and questions that Ehrenfels, in his highly ornate language, was addressing, and reveal some of the fundamental topics on which these pre-Mendelian scholars were focusing. Ehrenfels’s papers reveal that in Brno, “scientific naturalists”, as they sometimes called themselves, had been at work for decades addressing questions about biological inheritance in the animal and plant kingdom.

## 2. Biogeography and Publishing Activity

We know little of Baron Ehrenfels’s life. Josef Michael [24] (pp. 711–712) and Johann Markus (or a mixture of the two; see [25] (pp. 155–157); [26] (p. 7)) are used by historians as his first names. He was a Lower Austrian landowner, born on 9 March 1767 in either Retzbach or Zwettl, near Vienna, and he was called by his civil name “Judtmann”. In 1790 he married Countess Magdalene Louise Schonburg-Roschburg (1762–1833), taking the name “Ehrenfels”, and from then on held the title of baron. Their son was Carl Heinrich von Ehrenfels, whose own grandson was to be the influential philosopher Christian von Ehrenfels (1859–1932), one of the founders and precursors of Gestalt psychology [25] (pp. 155–157).

**Figure 2:**
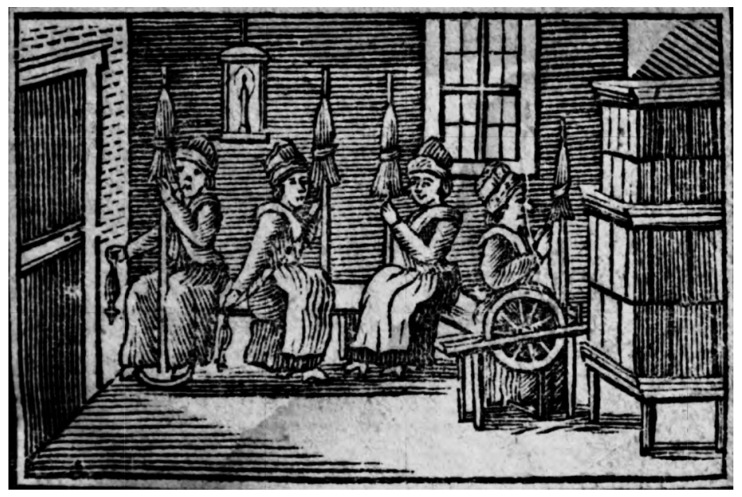
Title page of Ehrenfels’s book *Lessons for housemothers in their business* (*Der Erdmuthe Hülfreichinn Unterricht für Hausmütter in ihren Geschäften*), published in 1807. The engraving depicts women making yarn from wool with a spinning wheel. Image courtesy of the Austrian National Library, Vienna.

In addition to being an educated farmer in his day, Ehrenfels was also a well-read thinker interested in the arts and literature, and wrote extensively on agricultural subjects. “The cultivation of fine wool” [1] was a goal he pursued as a member of the Monarchy’s administration. He was regarded as one of the most creative and well-educated agricultural scientists of his time. His flowery and well recognizable writing style and his knowledgeable and astute dedication to his cause were unparalleled in the history of MAS in Brno. In spite of his neglect by historians, he deserves credit as the first in Central Europe to bring attention to the notion that a sheep breeder’s purpose should extend beyond merely producing a large amount of wool: it should also focus on genetic intervention (*genetische Einschreitung*) [1] (p. 140). In 1808, he wrote a book titled *Higher Sheep Breeding*, in which he emphasized the significance of selective sheep breeding and argued that the only sound breeding practice was uniqueness [27] (Figure 2). A staunch supporter of artificial selection, he wrote and spoke out against the sheep breeding methods of Albrecht Thaer (1752–1828). Ehrenfels [28] concluded that the environment had a definite influence on flocks. He was credited with discovering a cure for foot-and-mouth disease (FMD) in veterinary medicine at the time [29] (pp. 659–663).

In addition to animal health, Ehrenfels was equally passionate about apiculture [30,31]. He played a pivotal role in initiating the beekeeping industry in Austria. Due to a lack of skilled and enthusiastic beekeepers, his ideas to distribute beekeeping among the Habsburg Empire did not find immediate success. As a result, Ehrenfels built a large apiary in the Viennese Theresianum (or Theresian Academy) and gave public lectures on beekeeping [24] (p. 711). Later, together with Georg Rohrmoser, he also created in Brigittenau an apiary consisting of 150 beehives; this served as a public practical school for bee enthusiasts [32] (pp. 704–708), [33]. During the Napoleonic Wars, it was nearly destroyed, which prompted Ehrenfels to purchase properties in the area above the Mannhartsberg and establish a higher institute for beekeeping in Brunn am Wald and Allentigschwend. Together with Antal Nyáry (1803–1877), he also set the norms and regulations of the Budapest-based Hungarian Sheep Breeding Society [34]. The SBS of Brno, where he was a member till his death in 1843, published many of his essays during the 1800s. In addition to his practical activities, Ehrenfels was a prolific writer and published several books under the pseudonyms “Erdmann Hillfreich” and “Judtmann” [35] (p. 102). The topics of these works included diseases of farm animals [36], fruit trees [37], meadow and forage cultivation [38], sheep breeding [27,39,40,41], and agricultural economics [42]. As an active member of MAS until his death in Untermeidling on 9 March 1843, he developed his own theories on the role of a genetic force (*genetische Kraft*) in the heredity of desired traits in sheep breeding [42] (p. 137–138).

## 3. Selective Sheep Breeding in Central Europe

**Figure 3:**
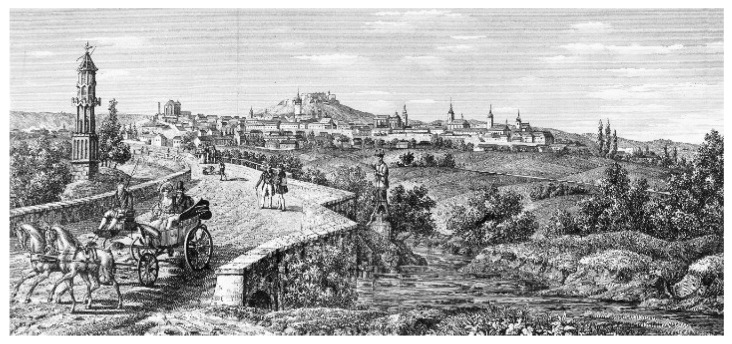
A view of early nineteenth-century Moravian Brünn (modern Brno, Czech Republic). Image courtesy of the Austrian National Library, Vienna (ALB 10734).

Brno’s early nineteenth-century sheep breeding debates were interwoven with philosophical and political debates on the nature of heredity [43]. Due to Spain’s exportation of fine wool from its Merinos or “noble sheep” during the Napoleonic Wars, quality wool from this breed became scarce in the years following 1800 [44] (pp. 24–56). It was largely factory owners, philanthropists and intellectuals, animal breeders, and natural scientists who were interested in wool enhancement in Central Europe’s private learned organizations. By helping each other, they aimed to create enormous volumes of fine wool in a short time to aid the army fighting on the battlefields. The need for an organization dedicated to only sheep breeding was pressing [45] (p. 183). Christian Carl André (1763–1831) founded the Sheep Breeders’ Society (SBS) in 1814 [46] (pp. 93–111). The full name of the society was the “Association of Friends, Experts and Supporters of Sheep Breeding for the achievement of a more rapid and more thoroughgoing advancement of this branch of the economy and the manufacturing and commercial aspects of the wool industry that is based upon it” (*Verein der Freunde, Kenner und Beförderer der Schaftzucht, zur noch höheren, gründlichen Emporhebung dieses Oekonomie-Zweiges und der darauf gegründeten, wichtigen Wollindustrie in Fabrikation und Händel*). In replicating The Society for the Improvement of British Wool established in Edinburgh in 1791, the SBS was the first animal breeding association on the European continent. It functioned as an independent branch of the MAS and was a merger between the “Society of Agriculture and Liberal Arts” (*Gesellschaft des Ackerbaues und der freien Künste*) and the “Moravian Society of United Friends for the Advancement of Nature and Homeland Studies” (*Gesellschaft der vereinigten Freunden zur Beförderung der Natur- und Vaterlandskunde in Mähren*) initiated by C. C. André [47] (p. 199). The term “natural science” in the society’s name symbolized the new approach laid down in its founding document, which aimed to study and understand nature as the “real” world [48] (p. 180). Consequently, the Moravian city of Brno (Figure 3), sometimes known as the “Moravian Manchester”, became a significant industrial center for wool manufacturing in the Habsburg Empire’s socioeconomic and ethnically varied social and cultural landscapes [49] (p. 17).

Central Europeans traveled to England, where new varieties of crops and farm animals became popular as breeders adopted novel methods for plant and animal improvement [50] (p. 12). For example, the New Leicester sheep raised by Robert Bakewell (1725–1795) demonstrated how enlightened breeding could result in “improved” breeds. Sheep breeding had been refined by Bakewell, who improved animal development rates and boosted tissue compositions for practical purposes while requiring little food consumption [51] (p. 83). Using inbreeding (“breeding in-and-in”), Bakewell determined that “seed” had a greater influence than climate on an animal’s physical appearance. In Moravian lands, consanguineous pairing was rejected on religious grounds and only a handful of progressive breeders such as Ferdinand Geisslern (1751–1824) of Hoštice, known as the “Moravian Bakewell”, and Count Imre Festetics (1764–1847) of Kőszeg, known as the “Hungarian Geisslern”, applied this method, rejecting incestuous taboos [52]. For Central European sheep breeders, evidence from artificial selection (*künstliche Zuchtwahl*) proved beyond a doubt that even characters considered non-essential by naturalists could be stably transmitted. Applying inbreeding resulted in more predictable heredity, and the breeders became interested in formulating questions about the very nature of this subject. Bernhard Petri (1767–1853) was convinced that, without mentioning or writing about it, the Spanish breeders had developed a genetically fixed race (*genetisch befestige Rasse*) of Merinos by allowing random variations to persist in the offspring through selective inbreeding [53] (p. 10). They realized that a merely noble flock can be elevated to what they called a “pure race” with care and attention by avoiding mixing alien blood and, through an appropriate control of pairings, bringing together specific characteristics of body build and wool to be transmitted to the progeny and preserved to the same extent. They observed that something constantly unique (*constant originelles*) arises, fixed in the organizational structure of living beings derived entirely and solely from pure-blood relatives (*aus lauter Blutsverwandten hergeleitete*) [54] (pp. 6–7).

This was then the big secret of Bakewellian breeding: match the parents based on their characteristics, engage in rigorous selection, and fix the type through inbreeding. To achieve racial stability, the solution was individually controlled matings (*Sprung aus der Hand*), and experience had shown that, even in the race flock, selective breeding was necessary.

## 4. The Problems of Heredity: Climate and Generation

The development of attitudes around inheritance was gradual. Progress in sheep breeding was examined critically at the annual meetings of SBS, with Ehrenfels actively involved. Members had come to recognize three reasons for faulty heredity: (1) environmental factors, (2) disruption resulting from the crossing of animals with differing essential features, and (3) sports or biological saltations. The solution had to be discovered through selective breeding in order to limit variability regardless of its source. As faith rose in the efficacy of selection, not just to retain but also to enhance desired attributes in sheep, more emphasis was placed on producing crosses to expand the diversity on which selection might work in novel ways. It was hoped that inbreeding would result in an improved breeding population, from which the quality might be elevated to new heights by the careful mating of males and females, and, thus, the disruptions in heredity caused by crossing might be managed and utilized.

**Figure 4:**
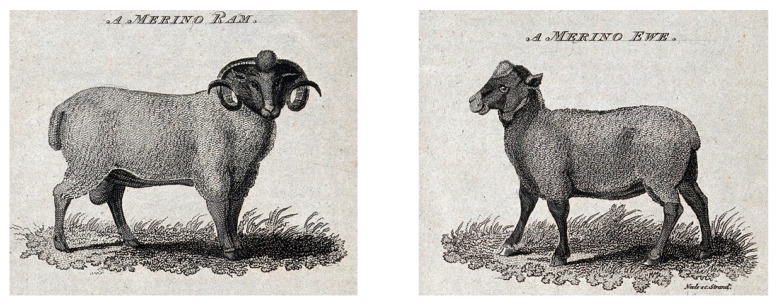
Stipple engraving by James Joshua Neele (1830). During the Napoleonic Wars, fine wool was obtained from Merinos, which were often termed “noble sheep”. The prolonged war prevented the importation of Merinos from Spain into the Habsburg Empire. The shortage of raw material for the textile industry was alleviated by breeders in Central Europe using inbred wool from local sheep breeds. Image courtesy of the Wellcome Library (no. 40111i).

From the beginning, Ehrenfels strongly rejected inbreeding. He stated that inbred bastard sheep arising from consanguinity are indisputably harmful to breeding [28] (p. 91). Ehrenfels believed the essence of animal organization was found in the climate, which was responsible for the formation of sheep, which was then echoed in descendants. In other words, constancy in inheritance was a direct effect of the climate. The opinion of Ehrenfels changed through a debate with Imre Festetics over his inbred Mimush sheep (Figure 4). Opposed to Ehrenfels, Festetics claimed that inbreeding was not harmful. Festetics formulated the “genetic laws of Nature” (*die genetischen Gesetze der Natur*) and the “fundamental laws of organic functions” (*Grundgesetze der organischen Funktionen*) to mitigate the Ehrenfels’s concerns about inbreeding [55,56]. Festetics maintained that generational changes observed in farm animals, plants, and humans are the product of scientific rules [57]. Festetics scientifically deduced that organisms inherit, rather than acquire, their features. He identified recessive traits and innate variation by hypothesizing that qualities from previous generations may re-emerge in later generations and that organisms could generate offspring with distinct characteristics. Festetics realized that inbreeding should be paired with careful selection. His discoveries are a significant antecedent to Mendel’s theory of particle inheritance insofar as they mark the shift of heredity from a myth to a scientific discipline in the twentieth century by giving a key theoretical underpinning for genetics [52].

The organic and genetic laws of Festetics based on empirical observations of the biological phenomena of heredity were recognized by Ehrenfels, who began using the phrase genetic (*genetische*) in a hereditary context in his writings and later addressed the questions of how much variety and constancy there is within animals with particular emphasis on sheep [1,42,58] (see Supplementary Material S1).

## 5. Variation and Constancy in Nature

Members of the SBS attempted to explain how nature develops new species of animals and plants through forces beyond human control, as well as how breeders regulate the reproductive process and implement modifications through crosses. Towards the end of his life, Ehrenfels began to formulate theoretical parallels between breeding and natural history in his last works. Variability and stability, according to him, were two sides of the same coin, stemming from the “genetic force, the mother of all living things” [1] (p. 130). In this sense, he distinguished between race (*Rasse*), which may be stable, and variation (*Varietät*), which could change through time and from generation to generation. Ehrenfels claimed that such variety is the basic mechanism by which life arises from the dead chaos of matter [42] (p. 137). In accordance with the philosophy of Festetics, he termed this process genetic mixing (*genetische Vermischung*), by which he meant the mixture of variable features in constant races [1] (p. 137). He argued that living organisms cannot defy the force of organizing components because their development is constrained by “genetic boundaries” [1] (p. 134). In his opinion, races have characteristics resulting from the climatic–genetic manifestation of the outward and interior organization of creatures [1] (p. 139). Therefore, deviations may return via genetic mechanisms in mating generations, which might be used to facilitate the efficient and rapid alteration of forms in breeding [1] (p. 139). Every individual is different from another conspecific, yet there is constancy within limits; in modern terms, they belong in a species, as recognized by aspects of their morphology. Ehrenfels saw parallels between domestic animals created through human culture and the natural–historical division of animal beings according to Buffon. Domestic animals are the products of artificial crossings, which over time developed into breeds; meanwhile, variations in nature genetically advanced (*genetisch fortbildet*) over time into species.

Ehrenfels [1] agreed with Albrecht von Haller (1708–1777), who in his preface to Buffon’s *Natural History* stated that no animal is entirely similar to another in its internal structure; they differ in the course of their nerves and veins, as in humans, so many millions of times that one can hardly find individual cases in which they agree. Ehrenfels asked: if the limited eye of the dissector finds this difference, how many more of its differences must the invisible force of nature express? He continued: if no two leaves of a tree are quite alike, they nonetheless remain leaves of one tree and express their genus so definitely and firmly in their form that no confusion is possible for the botanist. He also drew a parallel with sheep, which according to him were descended from the mouflon in many different forms, yet between all breeds distributed worldwide belong to the same genus. Equally as unexpected as the extent to which Ehrenfels allowed the alteration of natural forms is his emphasis on a fixed and unalterable number of species or types. When Ehrenfels pondered progressive development, he viewed nature’s evolution as the realization of a predetermined blueprint. For him, variation was limited by breeding within the corresponding animal varieties.

Ehrenfels’s reliance on species stability was founded on three notions advocated to varying degrees by German intellectuals such as Karl Friedrich Kielmeyer (1765–1844) and Karl Christian Gottlieb Sturm (1781–1826). The first principle, which stipulates that all members of a species are capable of producing fruitful progeny with one another, was the adoption of Buffonian principles. This concept of the creation of viable offspring allowed for a vast variety of modifications without compromising the stability of the total number of species. This is because it prevented the possibility of species transitions, which would have indicated the possibility of species evolution. Although there were growing questions about the applicability of this concept among other members of MAS in Central Europe, in the context of hybridization research (see later [2]), the infertility of mules to create fruitful offspring amongst themselves served as a case study supporting the idea [59].

The second principle suggested that there are a variety of stable animal types that may be modified based on an equilibrium or economic distribution model:

*“[…] all these creatures will reproduce constantly if they are mated and multiplied among themselves. Their fixed type will defend itself for centuries even against the powerful climate and remain what it finds, like the deer in the forest, so long as genetic force does not intervene and varieties are forced by mating with other strains and breeds. All animals which are not compelled by force or necessity to mate with other species, even the lascivious sparrow and the goat, remain constant, unchanged, for thousands of years, and disdain to mate with anything but their own kind*

[1] (p. 130).*”*

According to this basic idea, the increased growth of one organ within an organism coincides with the decreased development of another organ within the same organism. According to this equilibrium concept, the total number of modifiable organs within a given type remains constant. Despite having exceptional modifiability, there is no transfer between types. Breeding outside the corresponding type caused variation assisted by the environment. Climate, or (more broadly understood) the external environment, had an effect of the appearance, or in modern terms, phenotype, of an organism; but where does variation appear first? Ehrenfels seems to believe that animals were organized hierarchically, with essential bodily parts located more internally with respect to less essential parts located more peripherally. The effects of climate on the appearance of organisms were first seen on the outermost characters. Then, when nature acted upon the *innermost parts*, races were formed:

*“[o]nce nature has accomplished this change in the solid parts, it remains just as faithful to this newly created type as it did to the original, which had long been defended. Thus, she finally forms races, not only changed externally in skin and hair, in feathers and horns, but in all solid inner parts, which finally become organically solid, constant and hereditary from generation to generation and can no longer be modified by climate and external influence alone, but only by inner genetic force and mating*

[1] (p. 140)*.”*

By making the development of solid parts dependent on accomplished changes, Ehrenfels introduces the *genetic force* that is bound to a space but, within this space, exerts itself freely. While acknowledging freedom to an extent, Ehrenfels stressed that nature has its fixed courses, protecting its inner structure and directing the foreign influence first to the outer parts. In a later passage, he referred to the principle according to which nature distributes its powers within described limits. By this, he meant that even a central force governs within limits, for only food and reproduction, as living forces, express themselves more intimately and come to modify the solid parts definitively.

The third principle serves as a foundation for the second. This genealogical concept in Ehrenfels’s theory identifies the link between distinct changes in one species as one of descent. Thus, all individuals of a species are presumed to have descended from a single origin, one species pair, or a single progenitor. The common ancestor binds all variants together and sets them apart from their offspring descended from different ancestors. Common characteristics do not establish unity; rather, it is a shared ancestry that does so. This raises the question of how the origin should be imagined. The transformation of one race into the other in Ehrenfels’s framework depends on the permanence of *genetic force*:

*“[…] even the genetic force, the mother of all formations on earth, takes the climate under its peaceful or friendly power and lets come into being in the plant and animal kingdom, modified only with a visible finger, what can live under its cooperation*

[1] (p. 139).*”*

The progenitor, and the line originating from it, is able to change through the modifying effect of an inner power. Having shown how one race might be converted into another, Ehrenfels and his contemporaries wondered whether human selection and interference in copulation could permanently alter races, species, and ultimately nature. Breeders were confident of transforming a population via breeding. This shift from a climate-oriented theory of reproduction and modification of organic body parts through influence (*durch genetische Einwirkung*) thus coincided with the conviction that genealogical development depends on parental contribution [58] (pp. 3–4). In the context of breeding efforts, this renewed focus on the view that parental characteristics blend (*verschmelzen sich*) in progeny [60]. A previous theorem suggested that species transformation takes place via inborn components (*theils angeboren*) [55] (p. 9–10).

In this respect, Ehrenfels understood the transmission of parental traits in mechanistic terms, under the influence of a generative force. Ehrenfels believed the *genetic force*, defined like Blumenbach’s formative drive (*nisus formativus* or *Bildungstrieb*, see [61]), interacts with influences coming from the environment, both climatic and nutritional. It was strongest in its effect upon matings of the same sort. Ehrenfels recognized the need to explain why progeny deviates from the parental pattern. In this respect he made it clear that, among all influences, the genetic is the strongest. How are different breeds formed? In the context of wool qualities in sheep, Ehrenfels admitted that “*this question is not yet a mathematically solvable problem* ”, showing awareness of what in modern genetics is known as a quantitative character probably influenced by multiple genes [62].

## 6. The Process of Human Breeding

When the discoveries made in animal breeding were studied in connection to human development, the subject of the influence of human interference in nature became most relevant. This was because of the potential implications of these observations. When it came to abilities that have traditionally been considered of spiritual origin—specifically those ones thought to elevate the human being above the animal state—the question of whether hereditary patterns observed in animal populations under the pressure of selective breeding could be valid for human beings was particularly explosive. The belief in the extraordinary position of humans had been firmly established by the Christian tradition for a very long time, and it was further strengthened by reference to what we today may refer to as intellectual capabilities. In spite of this, the widespread interest in sheep breeding that existed throughout Brno in the early nineteenth century led to the re-examination of the human–animal split, of the concept of a strictly hierarchical chain of Nature, and of hereditary aristocracy through the André affair (see [43,63]). However, according to Ehrenfels [1] not only animals but all organisms possess the *genetic force* whose influence on their appearance is greater than that of the climate. As he stated, plants, humans, and animals each absorb the effects of their climate in their own way and process them organically:

*“What effects the climate has on animal organizations is revealed even in man by the formation of his mind and body. Although we have no climatology, climate is less dependent on heat and cold, the sun’s rays and the angle of their incidence, often more on the proximity of the sea, the prevailing wind, the altitude and depth of the land, rain and steam, and is therefore often local*

[1] (p. 131)*.”*

Ehrenfels contrasted this idea with the experiments of German chemist Lorenz Florenz Friedrich von Crell (1744–1816). According to Ehrenfels, Crell’s experiments on the ability of plants and animals to produce and destroy heat showed that the climate is a factor in the development of not only the body, but also the mind. Crell’s experiments demonstrated how humans and animals can exist in zones that exceed the temperature of their blood as well as survive where even spirituous fluids freeze. However, these climates must exert a powerful influence on the organization of plants and animals, so that “*the tree of the south in Greenland becomes a creeping shrub, the short-haired dog a shaggy bear, the man, in Kashmir a model of beauty and symmetry, in the farthest north an Eskimo: who could deny this effect and explain it otherwise than climatically?*” Ehrenfels touched upon a very important subject in maintaining a gap of human habit transfer among generations. This was essential to firmly establish and to protect free will. The autonomy of moral abilities and judgements may be called into doubt if it were possible for biological traits to transfer not just physical characteristics but also intellectual capacities.

Immanuel Kant (1724–1804), like Ehrenfels, drew a parallel between skin tone and sheep wool, noting that they both depended on local conditions [64]. The English physician Caleb Hillier Parry (1755–1822) also made the parallel, believing that the color of sheep wool and human skin will alter depending on the climate [65]. The late 1810s saw Ehrenfels hold similar beliefs [28], but debates concerning sheep wool showed him to be mistaken. In light of the fact that many breeds or races retained their traits after relocation, he reworked his “climate” theory. More and more breeders realized that Merino sheep could be successfully bred in a variety of places. During the 1830s, Ehrenfels, following in the footsteps of Festetics [55,56], saw evidence of a strong connection between generations that pointed to a relationship between humans and offspring regardless of climatic or other natural effects. As Ehrenfels iterated, the “blood” of animal breeds and humans allowed them to adapt to different climates and soils.

This was in contrast to the then-prevalent doctrine of race constancy, which was based on the concept of pure blood in horse breeding and developed by the German horse breeder Johann Christoph Justinius [66]. Johann Karl Nestler (1783–1841), professor of agricultural and natural sciences in Brno [10], also emphasized that constancy, the strict inheritance of racial traits from parents to progeny without deviation, cannot be found even in free Nature [59]. Nestler separated heredity from the age-old mystery of generation because he did not understand the physiological foundation of generation and the function of each parent in the formation of the embryo. Ehrenfels appreciated these new ideas and stressed they should be elaborated “according to principles and rules” [58] (p. 2–4). Ehrenfels distinguished inherited foundations of forms from physiologically limited traits inherited as a potential influenced by conditions during raising; he concluded that the *genetic force* is similar to the electric currents of fire, but its influence on the organization of organismal body formation remained unknown [1] (p. 131). Like C. C. André [46] (p. 103), Ehrenfels treated heredity as a mechanistic force analogous to the forces of physics, which became a major obstacle for further investigations.

## 7. Concluding Remarks: Role of the Sheep Breeders in the Development of Genetics

The sheep breeders pushed research into the basis of inheritance particularly as this activity was economically important to the Habsburg Empire, and some of them were genuinely interested in the science. Ehrenfels tried to organize his experience in sheep breeding and other agricultural activities into a coherent body of knowledge with which to make sense of heredity as he knew it and, importantly, of the traits of wool that interested him. Yet, years later, as if admitting failure, Ehrenfels [58] declared that the science and art of breeding remain in search of fundamental principles. He stated that sheep breeding was struggling for principles and fundamentals and, for this purpose, urgently needed the help of other auxiliary sciences such as natural science, anatomy, and physiology to decipher an unknown truth from a known one. In what now reads like a prophecy, Ehrenfels [58] emphasized the urgency of constancy as the basis of pedigree formation. This emphasis on the constancy of the characters was proved in Mendel’s experiments.

Although most readers are aware of the 3:1 and other such Mendel’s ratios on peas [67], *Pisum*, most of our students are unaware that Mendel was unable to replicate his ratios in *Hieracium* [68] or *Phaseolus* [69] and *Mirabilis jalapa* [70]. This could explain why Mendel rightly appears not to have thought his ratios universally applicable, although Hartl and Orel [69] seem to disagree. At the point in the history of genetics when the ancient and nineteenth-century approaches to heredity meet, Ehrenfels’s publications provide us with an excellent starting point for further research into Mendel’s experiments as well as many other fascinating discoveries. We can only speculate about the history of genetics had Mendel worked with sheep.

## Supplementary Materials

The following supporting information can be downloaded at: https://www.mdpi.com/article/10.3390/genes13081311/s1, Material S1: Transcriptions and translations of selected writings by J. M. Ehrenfels on biological heredity and sheep breeding published in the journals edited by the Moravian Agricultural and Natural Science Society.
