# Supplementary material for: Themes of Biological Inheritance in Early Nineteenth Century Sheep Breeding as Revealed by J. M. Ehrenfels
Source: Genes (Basel). 2022 Jul 23;13(8):1311. doi: 10.3390/genes13081311 (PMC9332421; doi:10.3390/genes13081311)
Supplement: Supplementary file 1 [file genes-13-01311-s001.zip › genes-1795758-supplementary.pdf]

Supplementary Material S1.

English translations and transcriptions of selected writings by J.M. Ehrenfels printed Fraktur German about biological heredity and sheep breeding published in the journals edited by the Moravian Agricultural and Natural Science Society.

Table of Content

1. Ehrenfels, J. M. 1829. On breed, variety and constancy in the animal kingdom [*Ueber Rasse, Varietät und Konstanz in Thierreich*]. *Mittheilungen* 17: 129–134; 18:137–142.
2. Ehrenfels J.M. 1831. Continuation of the thoughts of Mr. Moritz Beyer about the Merino sheep [*Fortsetzung der Gedanken des Herrn Moritz Bayer über das Merinoschaft*]. *Mittheilungen* 18:137–142.
3. Ehrenfels J.M. 1837. Written addendum to the transactions of sheep breeders - meeting in Brno, May 10, 1836. [*Schriftlicher Nachtrag zu den Verhandlungen der Schafzüchter-Versammlung in Brünn, am 10. Mai 1836*]. *Mittheilungen* 1:2–4.

1.

Notices
of the Imperial and Royal Moravian-Silesian Society for the Promotion of Agriculture, Natural History and Regional Studies in Brno.

Nro. 17.

April 1829.

On breed, variety and constancy in the animal kingdom.
At the request of Baron Emanuel v. Bartenstein.
(S. Nro. 10 of the Mittheilungen l. J.)

In response to my outline of the history of Austrian high-fine sheep breeding, as an addition to the overview of European sheep breeding by J. G. Elsner,[1] I have received several inquiries, commendations, doubts and objections from domestic and foreign scholars and economists. Among all of them, that of Baron Emanuel v. Bartenstein, whom I have long held in high esteem as one of the most industrious and educated economists in Austria, encouraged me to explain myself in more detail about breed, variety and constancy with regard to animals. Baron v. Bartenstein has arranged for the principles I have outlined in this history to be selected for the customary debate at the meeting of the Sheep Breeders' Association in Brno in 1829. Several views and experiences will probably follow from this; for the purpose of which I also wish to contribute, since this subject so powerfully intervenes in the essence of the breeding of high-fine sheep and in the preservation of the German wool trade.

I do not wish to make use of the natural-historical division of animal beings into genus and species, nor to repeat what Buffon says about this; I wish to speak in a more practical way, applied to sheep breeding, only of breeds and varieties. Baron v. Bartenstein thinks that I have not made known the data which support my assertion that fine-wool sheep breeding, which has resulted from original strains or purebred sheep, could perhaps never be made constant, or only after human ages, for the reason that such seem to me to be lacking, and therefore this assertion of mine must be regarded as a private opinion and reasoning in the absence of proof.

The Baron further believes: that all our domestic animals are products of accidental crossings, which have developed into breeds through time and human culture: that the Negretti and Escurial strains (more specifically the Electoral strain)[2] are very closely related, that the subdivision of the Merinos into these two strains is merely literary, and that the offspring resulting from the mating of the two strains are breedable as children of one strain; under which premises, in my opinion, can varieties become consolidated in their breed-specific wool, and several other things, is what the Baron wishes me to answer. These statements, which are honourable requests from such a pen, in consideration of such an effective Moravian sheep breeders' association, have encouraged me to present what I know to say in an enlightening manner under the heading: Breed, Variety and Constancy in the Animal Kingdom, applied to sheep, and to reflect on the results of my principles expressed in the history of Austrian sheep breeding.

---

[1] See Oeconom. Neuigk. No. 85, 86, 87, 1620. II. Heft. 1829.

[2] There is no longer an Escurial strain. This was lost in Spain to the Negretti and was transformed in Germany by climate and culture into the Electoral strain.

Haller, in his preface to Buffon's Natural History, says that no animal is entirely similar to another in its internal structure; they differ in the course of their nerves and veins, as in man, so many millions of times that one can hardly find in individual cases in which they agree. **If the limited eye of the dissector finds this difference, how many more of its differences must the invisible force of nature express? But even if no two leaves of a tree are quite alike, they still remain leaves of one tree and express their genus so definitely and firmly in their form that no confusion is possible for the botanist.** So too the sheep, descending from the mouflon, in so many different forms of its breeds under which it appears on earth, is everywhere one and the same genus. But wherever it has followed man as a domestic animal, this breed of sheep has become acclimatized, subjected to its culture and the resulting favourable or unfavourable changes. What effects the climate has on animal organizations is revealed even in man by the formation of his mind and body. Although we have no climatology, climate is less dependent on heat and cold, the sun's rays and the angle of their incidence, often more on the proximity of the sea, the prevailing wind, the altitude and depth of the land, rain and steam, and is therefore often local. Crell's experiments on the ability of plants and animals to produce and destroy heat, as well as **Crawford's experiments on the ability of plants and animals to produce cold,[3] have demonstrated how humans and animals can exist in zones that exceed the heat of their blood and also survive where even spirituous fluids are made to freeze. However, these climates must exert a powerful influence on the organization of plants and animals, so that the tree of the south in Greenland becomes a creeping shrub, the short-haired dog a shaggy bear, the man, in Kashmir a model of beauty and symmetry, in the farthest north an Eskimo: who could deny this effect and explain it otherwise than climatically? - – -**

What climate may be, we also know only in a human way. **Not the sensory perceptions alone, air, water, sustenance, etc., form the climate; a great storehouse of other invisible forces combines with them. The electric current of fire, this mighty being whose animal influence is as yet unknown to us, who calculates its existence and how the animal body receives and processes it?** We live on the breath of air, but who can tell us what the balm, our food of life, is in it? - What Hippocrates said in *De aere, locis et aquis*; what Gmelin said about the new discoveries in the air; what Gaubins said as a physician and pathologist; what Montesquieu said as a legislator; Castillon and Falcauer as jurists; what Wilsen said as a naturalist; what Linné said as a natural philosopher; even Baco in his history of revolutions and more recent criminal judges say about the climate and its influence, all this must be put together in order to recognize the power of this conflict, what climate means. **Even the genetic force, the mother of all formations on earth, takes the climate under its peaceful or friendly power and lets come into being in the plant and animal kingdom, modified only with a visible finger, what can live under its cooperation. Plants, men and animals each absorb the effects of their climate in their own way and process them organically. Thus, the Negro in Africa, the Eskimo in Greenland, in ancient Greece the living pattern image of Apollo and of Venus.**

The sheep in Spain and at the Cape of Good Hope are effects of the climate; there it must drag its fat tail away on a small wagon; in Germany its Merino wool is refined to a fineness which in the Electoral sheep wool, especially with stable fodder culture, often shows only fluff under feathers. **But here, too, nature observes her fixed course: she protects her inner structure and directs the foreign influence first to the outer parts. The most conspicuous varieties of the human race are limited to skin and hair; in the case of sheep, to skin and wool. Only food and reproduction, as living forces, express themselves more intimately and finally modify the solid parts definitively.** It is difficult to dissuade nature

[3] *) Phil, transact. Vol. 71. pg. 2 XXXI. Since it is not a matter of scholarship here, but rather of guarantors for my propositions, so let me be permitted citations only for the most important information. It does not sit well, I know it, and it does not inspire much confidence to adorn oneself in economic negotiations with citations that attest to more erudition than practice.

from the type it has once formed. When nations shape their heads for centuries, pierce their noses, lengthen their ears, nature ignores this external force and, through genetic power, long reshapes the original body type from within. But if she had to change once by continued millennial onslaught by climatic force, yes then she changed conformably all single parts to the whole. **In his comparative anatomy, Sömmering has given us a striking insight into the physical difference between the Moor and the European. Nature, not art, has flattened the Negro nose.** It is a consequence of the altered formation of his skull as a whole, even of the spinal cord and its course. Once **nature has accomplished this change in the solid parts, it remains just as faithful to this newly created type as it did to the original, which had long been defended. Thus, she finally forms races, not only changed externally in skin and hair, in feathers and horns, but in all solid inner parts, which finally become organically solid, constant and hereditary from generation to generation and can no longer be modified by climate and external influence alone, but only by inner genetic power and mating.**

Put the Moor in Europe, says a great man, Herder, he remains what he is, but marry him to a white woman, and a generation will change what centuries in the pale climate would not have done.

According to this premise, of which every word finds its guarantee in nature and in the depository of its purest priests, in the first authorities of true literary greatness, it is easy to express in just a few words: What is a breed in the animal world, is a breed among sheep. A breed is, among the types, a strain that is so conformably and constantly and firmly formed in its external and internal, in its solid and fluid parts, that **it maintains and reproduces itself conformably and constantly by its inherent genetic power alone, even under external temporal counteractions.**

A breed, as evidence, reveals itself by its solid formation, e.g., the canary among birds, the wolf among the wild, the pug among the tame breeds of dogs, the Negretti sheep, the Tyrolese cow among the many types or varieties of its kind, the horse of Arabia, the Moor in Africa; and in the vegetable world, the wild apple tree. - – -

All these creatures will reproduce constantly if they are mated and multiplied among themselves. Their fixed type will defend itself for centuries even against the powerful climate and remain what it finds, like the deer in the forest, so long as genetic force does not intervene and varieties are forced by mating with other strains and breeds. **All animals which are not compelled by force or necessity to mate with other species, even the lascivious sparrow and the goat, remain constant, unchanged, for thousands of years, and disdain to mate with anything but their own kind.** Not so the tame dog, which seldom finds like for mating, and therefore, like the European in Africa, unites with the race of blacks.

Varieties come to be (once again evidence) when the canary mates with the siskin, the wolf with the mastiff; the pug with the greyhound; the Tyrolese cow with the Swiss animal; the Negretti with the Electoral sheep; the Arabian with the English horse; the Moor with the European; the wild apple tree with the graft of the noble strain. All these varieties, however, are without constancy, merely ephemeral creatures, not guaranteed by their inner organization, merely endowed with the temporal ability to assimilate nobler things, and destined to reproduce among themselves, as they go back to their, and always to the most firmly formed, original line.

Thus, for several thousand years, the core of the apple trunk, refined by grafting, cannot yet do without the grafting or bud grafting of the noble strain in the case of repeated cultivation. The young of canaries and siskins, become siskins again, as soon as they continue to reproduce among themselves. The wolf becomes a dog, or vice versa; the Tyrolese cow, from crossed descendants with the Swiss animal, becomes again a Tyrolese cow, in continued mating of the resulting hybrids; the middle sheep, resulting from mating the Negretti and Electoral sheep, become again Negretti in continued mating among themselves; and the children of the Moor with the European woman become at first mulattos, and, according as they live in this or that climate, from mulattos again entirely black or white men. This is the order of nature: who changes it, who denies it? - **Here nature has directed two streams of life together, from which the nascent**

creature bears the traits of both parents. Which genetic force now overcomes the weaker one will prevail in the future creature, and thus the wild apple strain devours the graft, the wolf the dog, the Negretti the Electoral strain. After these premises, shall I now speak particularly of constancy? Is it not already clear that constancy is understood to mean the most intimate organic development of the outer and inner animal into a breed, that it remains a condition of this constancy that this animal reproduces as a result of its organically developed inner and outer body, like the Moor with the Moor, whether in Africa or Europe - - How difficult and slow nature now approaches this change, how long She strives to push out on skin, hair, wool, and feathers what works for the transformation of its inner form; how she evidently fails to reproduce, where, as in the horse and the donkey, she is forced into a hermaphroditic form; this we must say loudly: how difficult it is for nature to establish constancy, and how indulgent I was to admit that the crossed strains, hybrids and middle sheep only need human ages to consolidate their constancy. Less to blame is my first statement that these sheep may never attain constancy. It is true that in Asia, which is endowed with the greatest organic warmth, as the ancestral home of most of our domestic animals, **nature has genetically mixed the bezoar with the ibex through the intensity of the mating instinct, and from the procreation of more distant species than horse and donkey, has produced the so fertile breed of our goats with even reproductive capacity.** But what succeeded on this earth, where the electric current of fire probably first poured the formative principle in this first formative state liberally onto the young earth, where it created and nourished the cleverest apes, the wisest elephants, the liveliest animals; where presumably the hybrid children of the horse and the donkey would also have attained the ability to reproduce, that we no longer have to expect in Europe and Germany, or in our ageing world in general. Our most tameable animals, the sheep, the goat, the dog, the cow, are animal species of the Asiatic art of taming and mating, of the favourable heaven and the first creative power.

Although the southern islands of Asia were still full of tamed animals: so in New Guinea and New Zealand only the dog and the pig were found; in New Caledonia the dog alone; and in all the vast America there were only two tamed animals that could rise from the mud of the new world to live usefully in human society. The stream of fire that had been poured out in Asia, and which promoted all formation, could be carried over Africa in a slow step to Europe, and finally to America. By the time the combustible principal poured down from all volcanoes to the small pebble, which still gives fire with every blow, into all vegetation, into the seas and into animal life, Asia was already ahead of the fruit of the great Creation. - What can be scientifically proved today in Europe by a multitude of experiments and experiences, that animal life is merely occupied with the processing of the fuel and that the whole living cycle of creation seems to be that the liquid is solid, the solid liquid, the fire developed and bound again the living forces limited with organizations and set free again that nature had worked out in Asia in practical ways, as in a chemical laboratory on a large scale; what succeeded there will never succeed in Europe, much less in America. There, even the constancy of introduced original strains in all solid parts will change sacredly, while Europe will only establish its hybrids with difficulty. That is why I do not even shy away from America's competition for its wools, only on the quantity of its middle-quality wools can it press heavily. A classic book, Zimmermann's Geographical History of Man and the Common Four-footed Animals, reports: that a European sheep at the Cape of Good Hope had a tail of 19 pounds. In Iceland it sprouts to 5 horns; in England among the Oxfordians it grows to the size of an ass, and in Turkey it is tabby. What will become of it in America and Australia remains to be seen. Our Electoral sheep has the most similarity with the sheep from Kashmir; this country has a temperate climate like Germany and the products of its wool are finer than those from Electoral wool.

Baron von Bartenstein, you may take it from what has been said that I do not lack data to substantiate my assertion that fine sheep that have resulted from crossbreeding can perhaps never be made constant, or can only be made constant through the ages. Nature speaks aloud, not in tongues, but in a pictorial language comprehensible to all men, in

analogies, as I have already translated them above into human words. My experiences, which I would like to cite here, sound partisan. The reason why I did not mention this data immediately, however, was out of consideration for the reading public because such abstract treatises are not always welcome in economic journals.

That, as Baron von Bartenstein believes, all our domestic animals are the products of accidental crossings, which have developed into breeds through time and human culture, is true and demonstrable. I myself said earlier that our best domestic animals are genera of the Asiatic art of taming and mating. Why should Europe not also have practised and understood this art? In the case of the sheep, Bakewell and Saxony gave us a new proof. The English horse is another. The Tyrolean cow has in its exterior and interior, in solid parts, in skin and hair such a solid type that it, almost like the Negretti sheep, devoured all other strains mated with it and in several generations can hardly be merged. Nevertheless, it seems to be one of the youngest of the mountain strains. But what belongs to the formation of the variety as a breed; the change of the whole inner organization, conforming to all outer parts, transferred into the genetic power with the indomitable giant arm of constancy, that is the task.

Whether, and in how long a time, nature organically styles the varieties grown according to our needs so that they become a solid structure from within and without, a race strain which, like the Moor, preserves itself as a Moor in all zones; or whether nature ignores our crossings, not unlike the distortion of human ears, noses and Chinese girls' feet, genetically reverses them and keeps their type constant – that is the question?

This transformation is difficult, as analogy and experience show us every day. But one, tired of the circumcision of the Jews for millennia, has already seen circumcised Jewish children come into the world. I believe in the possibility of bringing varieties to constancy, although my experience speaks strongly against it. The wheel of creation and of the individual creatures, says Herder, goes around without adding or taking away; it destroys and builds in the genetic barriers in which it was placed in the first creating period. The power of the elements is bound to the cycle of certain organizations from which they themselves cannot depart, as the planet cannot fall from its orbit.

(Continuation follows.)

Notices
of the Imperial and Royal Moravian-Silesian Society for the Promotion of Agriculture, Natural History and Regional Studies in Brno.

Nro. 18.

May 1829.

On breed, variety and constancy in the animal kingdom.
At the request of Baron Emanuel v. Bartenstein.
(Cont.)

Baron von Bartenstein is of the further opinion: that the Negretti and Escurial strains, i.e., today's Electoral sheep, are very closely related; that their division into two strains is merely a literary aid and distinguishing mark, and that the offspring resulting from the genetic mixture of the two strains are also breedable as children of one strain.
If I had the mind of Sömmering and his previous knowledge of anatomy, I would be able to explain and prove anatomically, as he did the physical difference between the Moor and the European, the physical difference between the Negretti and the Electoral Sheep: what coarser and finer differences exist organically in both bodies. But are these differences not already perceptible to the unaided eye? - The formation of the bone structure between Negretti and Electorals; the muscle formation on the chest, back, and neck; the bulging skin of the one and the thin coat of the other; the long and short neck; the different head and tail; does not everything clearly show that nature has already changed all the solid parts, made them different, as in the case of the Moor from the European, and in this form genetically perpetuates each species, i.e., constantly passes it on; and that this is not the case. i.e., constantly inherited; in short, that the conditions, apart from skin and wool, are fulfilled, which we presuppose in purebred animals, and which are so different and distinctly expressed in both strains, as the Arabian versus the English horse? - How much finer and more frequent may be the invisible differences, the branchings in nerves and veins, the organic heat substance in the nerve fluid, in the blood? - – -
I have only examined the skin and the hair bulbs with the microscope, and found such a great difference in number and structure, in the position and direction of one and the other, that I am amazed how nature, with such different organs, could prepare the same product, only finer or coarser, longer or shorter, in such a similar way.
The short, curly woolly hair of the Negro, in contrast to the long, simple hair of a Dutchwoman, is hardly based on more anatomical and physiological differences than the modified structure of the skin between Negretti and Electorals! The Negretti sheep has entirely the African character in wool and sweat, while the Electoral sheep is more the original formation of Asia, perhaps from the fortunate valley of Kashmir, and this alone reveals two such different origins, even from two different parts of the world? The Negro of Africa and his skin is always covered with an oily yellow sweat, which both colours his retina black and gives his skin the tone of a thick soft velvet. In Africa's solar heat, nature has boiled an oil within, which blackens the net under the skin, but keeps the skin itself soft and moist against the burning sun's rays. Is it different with the original Negretti sheep? An excess of yellow sweat, which moistens the skin and wool, blackens the exterior of the latter and settles on the epidermis, winds up, as in the Negro, the hair into curling wool, and from the quantity of this skin fluid luxuriantly alive, at last into a dense, closed, rich woollen fleece. The Electoral

sheep, on the other hand, from a more temperate beautiful country of origin and most similar to the wool of Kashmir, has finer skin organs, fewer hair bulbs, finer, less and white sweat, hence white wool, outwardly white fleece, and since the hair lives on the abundance of the fine sap, which climatically needs to press on the skin surface less, the wool of the Kashmir and Electoral sheep grows by influx of the inner oil, more long than densely stacked; more delicate and, according to the visible oil or sap, also finer, than on all other sheep flocks. Therefore, according to these natural signs, we cannot declare the Negretti sheep to be more closely related to the Electoral sheep than the Negro is to the white; both are human children of one species, as Negretti and Electorals are sheep of the same species; but just as blacks and whites are divided into different race strains, so also the two sheep lines are divided into highly different, organically well-developed breed strains.

When one reads Bernier about Kashmir: the country of origin for the Escurial sheep imposes itself involuntarily. In the midst of the highest mountains, lies the happy land, hidden as in the paradise of the world. Fertile and beautiful hills, enclosed by higher and higher mountains, the last one, striking into the clouds, is covered with eternal snow, moderates the climate and moistens the land. Eternal green covers the ground, and wild and poisonous animals are banished there. The people of this part of the world are the most well-educated, and their women are known for their beauty, intelligence, and wit, skilled in all arts and crafts, even poetry. The wool of the Kashmir sheep is fluff - similar to the fur of the hare, finer than that of the Electoral sheep. With the climatic modification in Spain and Germany, this Kashmir sheep is in physique and wool most similar. I believe therefore: that the Negretti sheep developed climatically in Africa, the Electoral sheep originally emerged in Asia.

If we wanted to now add our experience to these reasons of the most eloquent nature and compare the results of the crossing of the two strains with the constancy of their offspring, how convincing would the statement have to be: that Negretti and Elektorals are very distant and in themselves very differently organized types and strains of sheep, which therefore cannot inherit constancy in their mixed blood and offspring, because this is the aggregate of all unchanged parts of the body and not only of the wool.

Finally, Baron v. Bartenstein believes that it is more advisable to mate varieties that have already succeeded in wool than to mate defective lines, since the lines themselves, according to my statement, have defects in themselves, and in all cases, reason dictates that the noblest be united with the noblest. Here I am misunderstood. Nowhere did I state that the strains were absolutely defective animals, and that they had relative, economically calculated defects only in regard to wool production. On the contrary, I[4] literally said in the 4th principle: in the Electoral sheep the highest wool fineness, in the Negretti the highest wool quantity is organically formed, and in the 5th principle: Electoral strains, which are inbred according to the principle of wool fineness, finally turn into economically defective wool sheep, just as Negretti inbred according to the principle of wool quantity, finally become coarse wool sheep. In fact, according to my statement and even more so according to the expression of nature, the Electoral sheep appears to be the highest model of fineness, the Negretti sheep the highest ideal of wool quantity in Europe. The highest in these main characteristics, however, also denotes the most perfect, the most noble. By uniting these noblest, what Baron v. Bartenstein quite rightly states as a principle: to unite the noblest with the noblest, is indeed followed on its own. The alleged faults of the strains are calculated only economically, relative to money, but not according to properties, according to fineness and quantity of wool. - The defective animals that offend against both principles, against economy and constancy, are always only the varieties that are good against constancy today, become worse in the next generation, and finally become characterless if the original strains do not intervene in a regenerating way. Throughout the entire animal kingdom, the ignoble is the variety, the hybrid; the noble, the breed, the original. Who, then, unites the noblest with the noblest; I, who for breeding

---

[4] Oekon. Neuigk. S. 691.

wool sheep use the purebred strains, or he, who for this purpose mates the always changeable varieties? - The greatest error, and none punishes the future more, is the lack of constancy.
I only need to give the desired answer to the question: how, in my opinion, could varieties be formed into consolidated, constant sheep breeds?
This question is easy and difficult to answer, depending on whether we believe in nature, in experience and conviction, or in desires, convenience and the market clamour of the young sheep breeders; for this question is not yet a mathematically solvable problem.
If constancy is neither more nor less than the faithful imprint of the father in the son (with the exception of the unspeakable nuances), the Moor of Moors; if this constant development is only a characteristic of race, but race is the climatic-genetic expression of the organization, an organic transformation of all external and internal, solid and liquid parts, a conforming addition of the individual organs to the whole organism of an animal breed: what does the weak man need against the strong principle of creation but to follow it by analogy, and to use its hints for his own ends? - –
a) A continued stimulation of nature, whether by climate or external force, determines her at last to yield, and to modify the organization according to this influence. Since Moses introduced circumcision among the Jews, one has at last seen circumcised Jewish children come into the world in some specimens. The Moor is still born white; but already in some parts of the body gradually blacker. The mountain which Linnaeus conceived to be the mountain of creation, Pallas, in his travels found less occupied with wild constant original animals, and more in the middle of Asia, than at the Linnaeus height of creation. The Escurial wool from Kashmir, as well as the African Negretti wool, formed in Spain, and the former in Saxony by climate and culture to fixed modified breed strains: slowly far beyond a human age, is this way, but not without consequences.
b) More effective and faster is the transformation, like the consolidation on the genetic path of mating and procreation. Here two forms are immediately and quickly fused into one.
c) Culture may therefore only stimulate nature by repeated procreation to continue to create in the image of the ideal or the real Electoral sheep, and as often as a deviation of the model strain should occur, to force the threatening deviation to return by genetic means by new mating individuals, e.g., by having a sheep of too short fine wool mated with a sheep of longer fine wool and vice versa.
d). In this way, the law of external excitation for consolidation and the law of genesis for constancy are followed at the same time.
There are no other ways in nature and art. This is how Bakewell created his meat sheep, this is how the Electoral sheep survived in Germany, and this is how the English horse was formed firmly, constantly, from the mixture with the Arabian.
Therefore, according to my principle, sub 10, we must maintain the original strains in the greatest purity, in order to always be ready to intervene with them, in the case of varieties and fine wool sheep breeding, as soon as deviations, which we would like to see removed from our Electoral sheep, become apparent. Everything else, to avoid repetition, follows naturally from my principles. How soon, however, nature will allow us to refrain from our suggestions, how long and how often we will have to climb up and down the ladder of refinement through genetic intervention, when and whether a constant resulting from popular varieties chosen for our purposes and wool culture will finally become an unchanging type, a breed, for this my life was not long enough, My life has not been long enough for me to state with certainty a time or even the number of generations in which this could be achieved, my experience has always given me negative information about this, and so I take the liberty of ending here with continued principles, as I did in my historical sketch of Austrian higher sheep breeding, and I ask you to add to it what follows after 10:

11) From the pure doctrine of breed, variety, and constancy in the animal kingdom depends the progressive and static ennoblement of the highly refined sheep breed, and the continuation of the German wool trade.
12) Climate, culture and genesis, i.e., mating and procreation, have divided the lines of the sheep into strains and into breeds,
13) **Genetic mixing of the strains gave rise to many varieties.**
14) A breed is a strain which is thus consolidated, so firmly marked by all organs, and which is able to reproduce itself constantly under all counteractions.
15) Varieties are the product of the mating of two different strains or varieties. They do not have the inner, solid, organic imprint, so that they are able to reproduce constantly, and strike more or less back to their original strains.
16) The setbacks happen, according to the law of the strongest with the participation of the climate. In Europe the mulatto becomes white, in Africa black. The lambs of the Negretti and Electoral sheep strains become Negretti in the setback, because Negretti are more firmly typed than Electorals.
17) Constancy is the character formed by all that is able to reproduce itself constantly under all conditions, like the Moor with the Moorish woman in Europe.
18) Asia was probably the first, beautiful place of cultivation of humans and our domestic animals. What we succeeded in doing then and there, we will not accomplish in Europe, still less in America. Our best domestic animals are species of Asiatic culture under Asiatic skies.
19) Out of this originally Asiatic culture, strains are formed which, acclimatized, nature has conformably changed in their entire inner organization, and in all solid and liquid parts, and under the indomitable giant arm of constancy has formed into breeds in different parts of the world.
20) Under European culture, the Electoral sheep in Saxony and Bakewell's meat sheep in England have, in more recent times, with some temporary help, taken on the form of a breed.
21) It is difficult for nature to pass over into alien forms; it is even more difficult for it to give this new form constancy and the ability to reproduce. The power of the elements is bound to the cycle of certain organizations, from which nature itself can hardly depart.
22) The Negretti sheep is of African, the Electoral sheep of Asiatic origin, therefore very distantly related and like one were the Moor, the other the white, belong to two very different strains.
23) The Electoral sheep is the highest and noblest in fineness, the Negretti sheep, the highest and most perfect in quantity of wool. Their economic relative defects do not deprive them of organic perfection. He who pairs both strains unites the noblest with the noblest. He who pairs varieties unites the imperfect with the imperfect.
24) The biggest fault with highly refined sheep is the lack of consistency.
25) Constancy in varieties, can only be obtained slowly, and by constantly stimulating nature to conform to our ideal or choice.
26) The formation step of a variety to constancy is the fusion of two beings into one by mating and procreation.
27) The most effective assistance is given to this breed training when every deviation from the original is met with genetic intervention, e.g., by mating too short-wooled mothers with rams of longer wool and vice versa.
28). This intervention will be necessary until nature, sooner or later, ensures constant reproduction by itself.
29) Only original strains can genetically improve variations, and therefore they must be maintained in constant evidence.
30) Within what time nature, even under this stimulus and assistance, will form the variety into a breed, the changeable into constant, cannot, for lack of experience, and before nobody’s time, be stated.

31) As to wool: the principles for its just appreciation are less in the clear than the doctrine of constancy. The manufacturers are certainly technological wool connoisseurs for the manufacture; but few know wool on the sheep, and still fewer are wool connoisseurs for the breeding of sheep. Moro, famous as a cloth manufacturer, is to me a competent and expert judge in that he says: no wool is too long for him, if only it is fine.

32) The short wool is not selectable because of the fabrication, but because of the breeding. Nature perfects faster in the short hair. Not that it would give a more excellent cloth, I have seen Mr. von Moro manufacture a cloth from my longest Electoral wool already in 1812, which I am looking for in vain today. If the long thread has the fineness and perfection of the short, it is better to use than the short. On the contrary, short wool is not suitable for delicate colours, such as scarlet, where the tips must be cut off.

33) The short hair fell into the hands of the factory owners as accidentally as it did into the hands of the sheep farmer. Neither found the reasons a priori, both only the consequences a posteriori. Short wool serves as a convenience for the manufacturer in the manufacture of cloth: for the sheep breeder, the excessively short hair is detrimental to the quantity of wool. The manufacturer finds the short hair more balanced and perfect in the thread, less weathered in the tips; he therefore prefers it for the cloth manufacture. It is, however, detrimental to fine fabrics because of its less flattering feel and coarser grip, although it dyes more easily without removing the tips. The breeder notices in the short-haired breed and type the refinement faster, more carefree to procure and the buyers for wool and breeding livestock fashionably easier to attract. If one spins the wool thread of the short and long hair and compares them under the microscope: in the thread of the short hair there are more nodes as points of unification of the threads, which cover in the cloth, ripping machine and finish, but has a detrimental effect for durability and fine feel in contrast to longer wool. Short wool defects are easier to hide in cloth; short wools are not or are less suitable for nobler products, for fine-coloured cloths, for scarlet, for shawls, for very fine Merinos and progeny, for all cloths that are supposed to combine lightness with durability. - This and more, is Mr. Elsner's answer to the type of wool he praises in hybrid wool as the most excellent today and as a product as ephemeral in manufacturing as the short-wool sheep will be in breeding. In the middle lies the truth.

It is a well-known fact that more money flows in from abroad through sheep's wool than through the export of all other products. I leave it to the patriots and the few who are able or willing to appreciate the importance of this, to decide whether such a serious national affair, the foremost of the active trade, does not deserve a state institution, encouragement, support, since everything around us is hurrying and striving to wrest the Golden Fleece from Austria. Austria has broken the ice, had to resort to publicity in order to enlighten its inhabitants, and, by communicating to it, as here, the results of older experience, had to enlighten foreign countries as well. There is, however, a science apart from that already known, which can be used for a long time in great institutions, and which can secure us the advantage for a long, long time.

J. M. Freiherr v. Ehrenfels.

Mittheilungen
der k. k. Mährisch-Schlesischen Gesellschaft zur Beförderung des Ackerbaues, der Natur- und Landeskunde in Brünn.

Nro. 17.

April 1829.

Ueber Rasse, Varietät und Konstanz im Thierreich.
Auf Aufforderung des Herrn Baron Emanuel v. Bartenstein.
(S. Nro. 10 der Mittheilungen l. J.)

Auf meine skizzierte Geschichte der österreichischen hochfeinen Schafzucht, als Zusatz zur Uebersicht der europäischen Schafzucht von J. G. Elsner*)[5], habe ich mehrere Anfragen, Belobungen, Zweifel und Einwendungen von in- und ausländischen Gelehrten und Oekonomen erhalten. Unter allen hat mich die des Hrn. Baron Emanuel v. Bartenstein, den ich längst als einen der industriösesten und gebildetsten Oekonomen Oesterreichs hochschätze, ermuntert, mich über Rasse, Varietät und Konstanz im Thier-Aufforderung gemäß, näher zu erklären. Hr. Baron v. Bartenstein haben veranlaßt, daß meine in der skizzierten Geschichte aufgestellten Grundsätze bei der Versammlung des Schafzüchtervereins in Brünn Anno 1829 zur üblichen Debatte gewählet worden. Daraus werden wahrscheinlich mehrere Ansichten und Erfahrungen folgen; zu dessen Behufe auch ich bei zutragen wünsche, da dieser Gegenstand fo mächtig eingreift in das Wesen der hochfeinen Schafzucht und in die Conservation des deutschen Wollhandels.
Ich will keinen Gebrauch machen von der naturgeschichtlichen Eintheilung thierischer Wesen in Genus und Species, nicht wiederholen, was Buffon hierüber sagt; ich will auf mehr praktischem Wege, angewandt auf Schafzucht, nur von Raffen und Varietäten sprechen. Hr. Baron v. Bartenstein findet, daß ich die Data, welche meine Behauptung, daß aus Urstämmen oder Rasselschafen entfallene Feinwolschäfereien, vielleicht nie oder nur nach Menschenaltern konstant zu machen wären, aus dem Grunde, weil solche mir vielleicht zu fehlen scheinen, nicht bekannt gemacht hätte und daher diese meine Behauptung in Ermangelung von Beweisen als Privatansicht und Raisonnement, gelten müsse.

Hr. Baron glauben ferner: daß alle unsere Hausthiere Produkte zufälliger Kreuzungen seyen, die sich durch Zeit und Menschenkultur zu Raffen ausgebildet haben: daß der Negretti- und Eskurialstamm (beffer gefagt der Elektoraltanum*)[6] sehr nahe verwandt seyen, daß die Unterabtheilung der Merinos in diese zwei Stämme bloß literarisch bestehe, und auch die aus Paarung beider Stämme resultirenden Nachkommen als Kinder eines Stammes rassefähig seyen; unter welchen Prämissen nach meiner Ansicht, Varietäten zu konsolidierten Raisseschafen werden können, wünscht der Hr. Baron und so mehreres von mir beantwortet. Diese Aeußerungen, die mir aus solcher Feder, Behufs eines so wirksamen mährischen Schafzüchtervereins ehrenvolle Aufforderungen sind, haben mich ermuntert, das, was ich aufklärend zu sagen weiß, unter der Ueberschrift: Raffe, Varietät und Konstanz im Thierreich, angewandt auf Schafe, vorzutragen und in Resultaten meiner in der Geschichte der österreichischen Schafzucht ausgesprochenen Grundsätzen nachzufeuden.

---

[5] S. ökon. Neuigk. Nr. 85, 86, 87, 1620. II. Heft. 1829.

[6] Es gibt keinen Eskurialstamm mehr. Dieser wurde
in Spanien von Negretti verfehlungen und ward in Deutschland durch Klima und Kultur in Elektoralstamm verwandelt.

Haller sagt in seiner Vorrede zu Buffons Naturgeschichte: daß kein Thier im innern Bau dem andern ganz ähnlich sey; sie unterscheiden sich im Laufe ihrer Nerven und Adern wie im Menschen so Millionenfach, daß man kaum in einzelnen Fällen auffindet, worin sie übereinkommen. Findet diese Verschiedenheit schon das beschränkte Auge des Zergliederers: wie viel seinere Verschiedenheiten muß die unsichtbare Kraft der Natur ausdrücken? Allein wenn auch keine 2 Blätter eines Baumes sich ganz gleichen, so bleiben sie doch Blätter eines Baumes und drücken in ihrer Form ihre Gattung so bestimmt und fest aus, daß keine Verwechslung dem Botaniker möglich wird. So ist auch das Schaf vom Muflon abstammend in so verschiedenen Formen seines Geschlechtes, unter welchen es auf der Erde erscheint, doch überall ein und dieselbe Gattung. Doch hat sich dieses Schafgeschlecht überall, wohin es den Menschen als Hausthier gefolgt, akklimatisirt, seiner Kultur und der daraus erfolgten günstigen oder ungünstigen Veränderung unterzogen. Welche Wirkungen das Klima auf thierische Organisationen habe, das offenbaret sich selbst am Menschen an der Ausbildung seines Geistes und Körpers. Obschon wir keine Klimatologie haben, Klima weniger von Hitze und Kälte, den Sonnenstrahlen und dem Winkel ihres Auffallens, oft mehr von der Nähe des Meeres, vom herrschenden Wind, von der Höhe und Tiefe des Landes, von Regen und Dünsten abhängig, daher vielfach lokal ist, so haben doch Crell's Versuche über das Vermögen der Pflanzen und Thiere, Wärme zu erzeugen und zu vernichten, so wie Crawford's Versuche Kälte hervorzubringen*)[7] nachgewiesen, wie Menschen und Thiere in Zonen bestehen können, die die Hitze ihres Blutes übersteigt, und auch fortleben da, wo selbst geistige Flüssigkeiten zum Frieren gebracht werden. Daß aber diese Klimate auf die Organisation der Pflanzen und Thiere einen fo mächtigen Einfluß nehmen müf sen, daß der Baum des Südens in Grönland zum kriechenden Strauch, der kurzhaarige Hund, zum zottigen Bär, der Mensch, in Kaschemire ein Mus ster der Schönheit und des Ebenmaßes, im höchsten Norden zum Eskimo werde: wer könnte diese Wirkung bestreiten und sie anders als klimatisch erklären? – -

Was Klima sey, wissen auch wir nur auf menschliche Weise. Nicht die sinnlichen Wahrnehmungen allein, Luft, Waffer, Nahrung u. s. w. bilden das Klima; ein großes Vorrathshaus anderer unsichtbarer Kräfte, verbindet sich mit diesen. Der elektrische Feuerstrom, dieses mächtige, uns in seinem animalischen Einfluß noch unbekannte Wesen, wer berechnet dessen Daseyn, und wie es der thierische Körper aufnimmt und bearbeitet? Wir leben vom Hauch der Luft, wer enträthielt uns aber, was der Balsam, unsere Lebensspeise, in ihr sey? – Was Hippokrates de aire, locis et aquis; was Gmelin über die neuen Entdeckungen in der Luft; was Gaubins als Arzt und Patholog; was Montesquieu als Gesetzgeber; Castillon und Falcauer als Rechtsgelehrte; was Wilsen als Naturforscher; was Linné als Naturphilosoph; selbst Baco in seiner Geschichte der Revolutionen und neuere Kriminalrichter vom Klima und dessen Einfluß sagen, das alles muß man zusammenstellen, um die Macht dieses Conflikts, was Klima heißt, anzuerkennen. Selbst die genetische Kraft, die Mutter aller Bildungen auf Erden, nimmt das Klima unter seine friedliche oder freundliche Macht und läßt im Pflanzen- und Thierreich, nur mit sichtbarem Finger modifiziert, entstehen, was unter seiner Mitwirkung leben kann. Pflanze, Mensch und Thier nehmen jedes nach ihrer Art und Weise die Einwirkungen ihres Klimas auf und verarbeiten sie organisch. So entfiel der Neger in Afrika, der Eskino in Grönland, im alten Griechenland das lebendige Musterbild des Apollo und der Venus.

Das Schaf in Spanien und auf dem Vorgebirge der guten Hoffnung, sind Auswirkungen des Klimas; da muß es seinen Fettschwanz auf einem kleinen Wagen fortziehen, in Deutschland läutert sich seine Merinoswolle bis zu einer Feinheit, die im Elektoralfhaf, besonders bei Stallfutterkultur, oft nur Flaum unter Federn zeiget. Aber auch hier beobachtet die

[7] *) Phil, transact. Vol. 71. Pg. 2 XXXI. Da es hier nicht um Gelehrsamkeit, sondern um Gewährsmänner für meine Sätze zu thun ist, so wolle man mir Citationen nur für die wichtigsten Angaben erlauben. Es läßt nicht gut, ich weiß es, und es erweckt wenig Vertrauen, sich in ökon. Verhandlungen mit Citationen zu schmücken, die mehr Gelehrsamkeit, als Praxis beurkunden.

Natur ihren festen Gang: sie schützt ihr inneres Gebilde und leitet die fremdartige Einwirkung zuerst auf die äußern Theile. Die auffallendsten Varietäten des Menschengeschlechtes beschränken sich auf Haut und Haare; bei dem Schaf auf Haut und Wolle. Nur Nahrung und Fortpflanzung äußern sich als lebendigere Kräfte inniger und modifiziren endlich auch die festen Theile definitiv. Schwer ist die Natur abzubringen von ihrem einmal geprägten Typus. Wenn Nationen Jahrhunderte lang ihre Köpfe formen, ihre Nasen durchbohren, ihre Ohren verlängern, die Natur ignoriert diese äußere Gewalt und formt durch genetische Kraft, lange das ursprüngliche Körperbild von Innen her aus wieder. Wenn sie aber einmal durch fortgesetzten tausendjährigen Andrang durch klimatische Gewalt ändern mußte, ja dann änderte sie konform alle einzelne Theile zum Ganzen. Sömmering hat uns über die körperliche Verschiedenheit des Mohren vom Europäer, in seiner vergleichenden Anatomie, frappanten Aufschuß gegeben. Natur, nicht Kunst, hat die Negers-Nase geplattet. Sie ist eine Folge der veränderten Bildung seines Schädels im Ganzen, sogar des Rückenmarks und seines Verlaufes. Hat die Natur einmal diese Veränderung der festen Theile vollzogen, so bleibt sie diesen neu geschaffenen Typum eben so treu, als dem früher lange vertheidigten Original. So bildet sie endlich Rassen, nicht nur äußerlich verändert an Haut und Haaren, an Federn und Hörnern, sondern an allen festen innern Theilen, die endlich organisch fest, konstant und erblich übergehen von Generation zu Generation und nicht mehr durch Klima und äußere Einwirkung allein, sondern nur durch die innere genetifche Kraft und Paarung wieder modifiziert werden können.

Setzet den Mohren nach Europa, sagt ein großer Mann, Herder, er bleibt was er ist, verheirathet ihn aber mit einer Weißen und Eine Generation wird verändern, was Jahrhunderte das bleichende Klima nicht würde gethan haben.

Nach dieser Voraussetzung, wovon jedes Wort seine Gewähr in der Natur und in dem Depositorio ihrer reinsten Priester, in den ersten Authoritäten wahrer literarischer Größe findet, ist nur mit wenig Worten leicht auszusprechen: was Rasse in der thierischen Welt, was Raffe unterdem Schafe sey. Raffe ist unter Geschlechtern ein Stamm, der in seinen äußern und innern, in seinen festen und flüssigen Theilen so konform und konstant und fest ausgebildet ist, daß er sich durch seine in wohnende genetische Kraft allein, selbst unter äußern zeitlichen Gegenwirkungen konform und konstant erhält und fortpflanzt.

Raffe, als Belege, offenbaret durch seine feste Ausbildung, z. B. der Kanarie unter Vögeln, der Wolf unter dem wilden, der Mops unter den zahmen Hundsgeschlecht, das Negrettifchaf, die Tirolerkuh unter den vielartigen Abarten oder Varietäten ihrer Art, das Pferd Arabiens, der Mohr in Afrika; und in der Pflanzenwelt, der wilde Apfelbaum. – -

Alle diese Geschöpfe werden sich konstant fortpflanzen, wenn sie unter sich gepaart und vermehrt werden. Ihr fester Typus wird sich Jahrhunderte selbst gegen das mächtige Klima vertheidigen und bleiben was sie find, wie der Hirsch im Walde, so lange nicht die genetische Kraft einschreitet und Varietäten durch Paarung mit andern Stämmen und Rassen erzwungen werden. Alle Thiere, welche nicht durch Zwang oder Noth zur Begattung mit andern Arten gezwungen werden, selbst der wollüstige Sperling und die Ziege, bleiben Jahrtausende konstant, unverändert und verschmähen sich anders als mit ihres Gleichen zu paaren. Nicht also der zahme Hund, der selten Gleiches zur Begattung findet, und deshalb wie der Europäer in Afrika sich mit der Rasse der Schwarzen einiget.

Varietäten werden, (abermals Belege) wenn sich der Kanarienvogel mit dem Zeisig paart, der Wolf mit dem Fleischerhund; der Mops mit dem Windspiel; die Tirolerkuh mit dem Schweizertier; das Negretti- mit dem Elektoralschaf; das arabische mit dem englischen Pferd; der Mohr mit der Europäerinn; der wilde Apfelbaum mit dem Pfropfreis des Edelstammes. --Alle diese Varietäten find jedoch ohne Konstanz, bloß ephemere Geschöpfe, von der innern Organisation nicht garantirt, bloß mit zeitlicher Fähigkeit sich Edleres zu assimiliren, ausgestattet und bestimmt, unter sich fortgepflanzt, wie der zurückzugehen auf ihren und immer auf den festgebildetsten Urstamm.

So kann seit mehreren Tausend Jahren der durch Pfropfreis veredelte Kern des Apfelstammes, bei einem wiederholten Anbau, noch nicht das Pfropf oder Okulirreis des Edelstammes entbehren. Die Jungen der Kanarien und Zeisige,

werden wieder Zeisige, sobald sie sich unter sich weiter fortpflanzen. Der Wolf wird Hund oder umgekehrt; die Tirolerkuh wird aus gekreuzten Abkömmlingen mit dem Schweizertier, in fortgesetzter Paarung entfallener Bastarde wieder Tirolerkuh; die aus der Paarung mit dem Negretti- und Elektoralschaf entfallenen Mittelschafe, werden in fortgesetzter Paarung unter sich wieder Negretti; und die Kinder des Mohren mit der Europäerinn werden Anfangs Mulatten und je nachdem sie in dem oder jenem Klima leben, aus Mulatten wieder ganz schwarze oder weiße Menschen. Das ist die Ordnung der Natur: wer ändert, wer läugnet sie? – Zwei Ströme des Lebens hat hier die Natur zusammen geleitet, wovon das werdende Geschöpf die Züge beider Eltern au sich trägt. Welche genetische Kraft nun die schwächere überwindet, wird in künftigen Geschöpf vorherrschen und so verschlingt der wilde Apfelstamm das Pfropfreis, der Wolf den Hund, der Negretti- den Elektoralstamm.
Soll ich nach diesen Prämissen nun noch besonders von der Konstanz reden? Wird es nicht bereits klar, daß unter Konstanz die innigste organische Ausprägung des äußern und innern Thieres zur Rasse verstanden, es eine Bedingung dieser Konstanz bleibt, daß sich dieses Thier in Folge ihres organisch ausgeprägten innern und äußern Körpers, fortpflanze, wie der Mohr mit der Mohrinn, ob in Afrika oder Europa - - Wie schwer und langsam nun die Natur an diese Veränderung gehet, wie lange sie auf Haut, Haar, Wolle und Federn hinauszudrängen strebt, was für die Umstaltung ihres inneren Gebildes einwirkt; wie sie offenbar die Fortpflanzung verfagt, wo sie, wie beim Pferd und Efel, zur Zwittergestalt gezwungen wird; dieß muß uns ja laut sagen: wie schwer der Natur Konstanz auszuprägen wird, und wie nachgiebig ich war zuzugeben, daß die gekreuzten Stämme, Bastarde und Mittelschafe, nur Menschenalter zur Konsolidierung ihrer Konstanz benöthigen. Weniger zu tadeln ist meine erste Angabe, daß diese Schafe vielleicht nie zur Konstanz gelangen. Zwar hat die Natur in dem mit der größten organischen Wärme begabten Asien, als Stammort unserer meisten Hausthiere, das Bezoarthier mit dem Steinbock durch Heftig keit des Begattungstriebes genetisch vermischt und aus der Zeugung mehr entfernter Geschlechter als Pferd und Esel die so fruchtbare Rasse unserer Ziegen, selbst mit dem Fortpflanzungsvermögen entstehen lassen. Allein, was auf diesem erhalte, ein Erdrücken der Welt gelang, da wo wahrscheinlich der elektrische Feuerstrom das Bildungsprincip in dieser ersten Bildungsstätte zuerst freigebig auf die junge Erde goß, wo es die klügsten Affen, die weifesten Elephanten, die lebhaftesten Thiere erschaffen, und nährte; wo vermuthlich auch des Pferdes und des Esels Barfardkinder die Fähigkeit der Fortpflanzung erreicht hätten, das müssen wir in Europa und Deutschland, überhaupt in unserer alternden Welt nicht mehr erwarten. Unsere zähmbarsten Thiere, das Schaf, die Ziege, der Hund, die Kuh, sind Thiergattungen der asiatischen Bezähmungs- und Paarungskunst, des dazu günstigen Himmels und der ersten Schöpfungskraft.
Wenn die Südinseln Asiens noch voll gezähmter Thiere find: so fand sich in Neuguinea und Neuseeland nur der Hund und das Schwein; in Neukaledonien der Hund allein und im ganzen weiten Amerika waren nur zwei gezähmte Thiere, die sich aus dem Schlamm der neuen Welt aufschwingen konnten, in der menschlichen Gesellschaft brauchbar zu leben. Der Feuerstrom der in Asien ausgegossen, war und alle Bildung förderte, kann über Afrika in langsamem Schritt nach Europa, zuletzt nach Amerika. Bis sich da von allen Vulkanen bis zum kleinen Kiefel herab, der heute noch bei jedem Schlag Feuer gibt, das brennbare Prinsip in die ganze Vegetation, in die Meere und in das animalische Leben ergoß, hatte Asien bereits die Frucht der großen Schöpfung voraus. – Was man heute in Europa durch eine Menge Versuche und Erfahrungen scientifisch beweisen kann, daß sich das animalische Leben bloß mit der Verarbeitung des Feuerstoffes beschäftige und der ganze lebendige Kreislauf der Schöpfung der zu seyn scheint, daß das Flüssige fest, das Feste flüssig, das Feuer entwickelt und wieder gebunden, die lebendigen Kräfte mit Organisationen beschränkt und wieder befreit werden, das hatte die Natur in Asien auf praktischen Wegen, wie in einem chemischen Laboratorio im Großen ausgearbeitet, Was da gelang, wird nie in Europa, noch weniger in Amerika gelingen. Dort wird sich felbst die Konstanz eingeführter Urstämme in allen festen Theilen macht heilig verändern, während Europa nur die Confolidirung seiner Bastarde schwer finden wird. Darum ich auch für seine Wollen die Konkurrenz Amerikas

nicht scheue, nur auf Quantität der Mittel wollen kann es schwer drücken. Ein klassisches Buch, Zimmermann's geographische Geschichte des Meschen und der allgemein verbreiteten 4 füßigen Thiere, berichtet: daß ein europäisches Schaf am Vorgebirg der guten Hoffnung einen Schwanz von 19 Pfunden bekam. In Island treibt es bis 5 Hörner; in England im Orfordischen wächst es bis zur Größe eines Esels, und in der Türkei ist es getiegert. Was in Amerika und Neuholland aus ihm wird, stehet zu erwarten. Unser Elektoralschaf hat die meiste Aehnlichkeit mit den Schafe aus Kaschemire, dieses Land hat das gemäßigte Klima wie Deutschland und die Fabrikate seiner Wolle sind feingriffiger als aus Elektoralwolle.

Hr. Baron v. Bartenstein belieben aus dem Gesagten zu entnehmen, daß es mir nicht an Daten fehle, meine Behauptung: daß aus der Kreuzung entfallene Feinschafe vielleicht nie oder nur durch Menschenalter konstant zu machen seyen, zu begründen. Die Natur spricht laut, nicht im Zungenlaut, sondern in einer allen Menschen verständlichen Bildersprache, in Analogien, wie ich sie oben bereits in Menschenworte übersetzt habe. Meine Erfahrungen, die ich hierüber anführen möchte, klingen partheiisch. Warum ich diese Data aber nicht sogleich angeführt habe, geschah aus Schonung für das lesende Publikum, denn so abstrakte Abhandlungen in ökonomischen Blättern nicht immer willkommen sind.

Daß, wie Hr. Baron v. Bartenstein glaubt, alle unsere Hausthiere, Produkte zufälliger Kreuzungen sind, die sich durch Zeit und Menschenkultur zu Rassen ausgebildet haben, darin liegt Wahres und Erweisliches. Ich selbst sagte früher: daß unsere besten Hausthiere, Thiergattungen der asiatischen Bezähmungskunst und der Paarungswiffenschaft seyen. Warum sollte Europa diese Kunst nicht auch geübt und verstanden haben? Beim Schaf gab uns Bakewell und Sachse in einen neuen Beweis. Das englische Pferd ist ein anderer. Die Tirolerkuh hat in ihrem Aleußern und Innern, in festen Theilen, in Haut und Haaren einen so festen Typum, daß sie beinahe wie das Negrettischaf alle andern Stämme mit ihr gepaart, verschlingt und in mehreren Generationen sich kaum verschmelzen läßt. Gleichwohl scheint sie einer der jüngsten Gebirgsstämme zu seyn. Was aber zur Ausbildung der Varietät als Rasse gehörig; die Umänderung der ganzen innern Organisation, konform mit allen äußern Theilen übergetragen in die genetifche Kraft mit dem unbeugsamen Riesenarm der Konstanz, das ist die Aufgabe.

Ob und in wie langer Zeit die Natur die, nach unserm Bedarf angezogenen Varietäten organisch dahin stylisirt, daß sie ein festes Gebild von Innen und Aussen werden, ein Raffe stamm, der dem Mohren gleich sich unter allen Zonen als Mohr erhält; oder ob Natur unsere Kreuzungen nicht etwa wie die Verzerrung der Menschenohren, der Nasen und chinesischen Mädchenfüffe ignorirt, genetisch umkehrt und bei ihrem Typum konstant bleibt, das ist die Frage?

Schwer geht sie an diese Verwandlung, das zeigt uns täglich Analogie und Erfahrung. Doch hat man, der Beschneidung der Juden seit Jahrtaufenden müde, auch schon beschnittene Judenkinder zur Welt kommen sehen. Die Möglichkeit, Varietäten zur Konstanz zu bringen, glaube ich; obschon meine Erfahrung hart dagegen spricht. Das Rad der Schöpfung und der einzelnen Geschöpfe, sagt Herd er, läuft umher, ohne daß es hinzuthue oder wegnehme; es zerstört und bauet in den genetischen Schranken, in die es der erste schaffende Zeitraum gesetzt hat. Die Macht der Elemente ist an den Kreislauf bestimmter Organisationen gebunben, aus dem sie selbst nicht weichen, wie der Planet aus seiner Bahn nicht fallen kann.

(Der Beschluß folgt.)

Mittheilungen
der k. k. Mährisch-Schlesischen Gesellschaft zur Beförderung des Ackerbaues, der Natur- und Landeskunde in Brünn.

Nro. 18.

Mai 1829.

Ueber Rasse, Varietät und Konstanz im Thierreich.
Auf Aufforderung des Herrn Baron Emanuel v. Bartenstein.
(Beschluß.)

Herr Baron v. Bartenstein ist sernerer Meinung: daß der Regretti- und Eskurialstamm, d. i. das heutige Elektoralschaf sehr nahe verwandt seyen; daß ihre Abtheilung in 2 Stämme bloß ein literarisches Behelf und Unterscheidungszeichen, und daß auch die aus der genetischen Vermischung beider Stämme resultierenden Nachkommen, als Kinder eines Stammes rassefähig seyen.

Wenn ich den Geist Sömmerings und seine anatomischen Vorkenntnisse hätte: so würde ich wie er, über die körperliche Verschiedenheit des Mohren vom Europäer, die körperliche Verschiedenheit des Negretti zum Elektoralschaf anatomisch darthun und erweisen können: welche gröbern und seineren Verschiedenheiten in beider Körper organisch existiren. Doch find diese Abstände nicht schon dem unbewassneten Auge merkbar? – Die Bildung des Knochenbaues zwischen Negretti und Elektorals; die Muskelbildung an Brust, Rücken und Hals, die gewulstete Haut des Einen und das seine dünne Fell des Andern, der lange und kurze Hals, der verschiedene Kopf und Schweif; zeigt nicht alles deutlich, daß die Natur bereits alle festen Theile geändert, verschieden wie dem Mohren von dem Europäer ausgeprägt und in dieser Form jede Art genetisch fortbildet, d. i. konstant vererbet; kurz die Bedingungen, abgesehen von Haut und Wolle, erfüllet, die wir bei Kassethieren voraussetzen, und die sich in beiden Stämmen so verschieden und deutlich aussprechen, als das arabische gegen das englische Pferd? – Wie viel seiner und häufiger mögen die unsichtbaren Unterschiede, die Verzweigungen in Nerven und Adern, der organische Wärmestoff im Nervensaft, im Blute seyn? – -

Ich habe bloß die Haut und die Haarzwiebeln mit dem Mikroskope vergleichend, untersucht, und so nächtigen Unterschied in Zahl und Bau, in der Lage und Richtung des Einen und Andern gefunden, daß ich staune, wie die Natur mit so verschiedenen Organen, dasselbe Produkt, nur feiner oder gröber, länger oder kürzer, so ähnlich bereiten könnte. Dem kurzen, krausen Wollhaar des Negers, gegen das lange, schlichte Haar einer Holländerinn, liegen kaum mehr anatomische und physiologische Abweichungen zum Grunde, als dem modifizierten Bau der Haut zwischen Negretti und Elektorals! Das Negrettischaf hat ganz den afrikanischen Charakter in Wolle und Schweiß, während das Elektoralschaf mehr die Urbildung Asiens, vielleicht aus dem glücklichen Thal von Kaschemire und schon dadurch zwei so verschiedene Abkünfte, sogar aus zwei verschiedenen Welttheilen, offenbaret? Der Neger Afrika's und dessen Haut ist immer mit einem öligen gelben Schweiß bedeckt, der sowohl feine Netzhaut schwarz färbet, als seiner Haut im Griff den Ton eines dicken weichen Sammts gibt. In Afrikas Sonnenwärme hat die Natur ein Oel im Innern gekocht, das das Netz unter der Haut schwarz färbt aber die Haut selbst gegen die brennende Sonnestets weich und feucht

erhält. Ist es mit dem Original des Negrettischafes anders? Ein Uebermaß von gelbem Schweiß, die Haut und Wolle befeuchtet, das Aeußere derselben schwärzt und auf die Oberhaut absetzt, windet sich wie beim Neger das Haar in kräuselnde Wolle und von der Menge dieses Hautsaftes üppig lebend, endlich in dichtes, geschlossenes, reiches Wollvließ zusammen. Das Elektoralschaftent- gegen, aus einem mehr gemäßigten schönen Ursprungsland und der Wolke aus Kaschmire am ähnlichsten, hat seinere Hautorgane, weniger Haarzwiebel seineren, weniger und weißen Schweiß, daher weiße Wolle, äußerlich weißes Vließ, und da das Haar von den Ueberflusse des seinen Saftes lebt, der sich klimatisch weniger auf die Hautoberfläche zu drängen, braucht, so wächst die Wolle des Kaschemires und Elektoralschafs durch Einströmung des innern Oeles, mehr lang als dicht gestapelt; zarter und nach den sichtbar seineren Oel oder Hautsaft auch seiner, als auf allen andern Schafarren. Wir können darum, nach diesen Naturzeichen, das Negretti- mit dem Elektoralschaf nicht näher verwandt erklären, als der Neger zum Weißen; beide sind Menschenkinder eines Geschlechtes, wie Negretti und Elektorals, Schafe eines Schafgeschlechtes find; aber wie sich Schwarze und Weiße in verschiedene Rassestämme theilen: so theilen sich auch die beiden Schafstämme in höchst verschieden ausgezeichnete, organisch fest ausgebildete Rassestämme.

Wenn man Bernier über Kaschemire liest: so drängt sich das Ursprungsland für das Eskurialschaft unwillkührlich auf. Mitten im Schooß der höchsten Gebirge, liegt das glückliche Land, verborgen wie im Paradies der Welt. Fruchtbare und schöne Hügel, mit höheren und immer höheren Bergen umschlossen, ist, der letzte in die Wolken streikkend mit ewigem Schnee bedeckt, mäßiget das Klima und befeuchtet das Land. Ewiges Grün bedecket den Boden, und wilde, und giftige Thiere find da verbannt. Der Mensch dieses Erdenpunktes, ist der wohlgebildetste und ihre Weiber bei kannte Muster der Schönheit, geistreich, witzig, zu allen Handthierungen und Künsten, selbst zur Poesie, geschickt. Die Wolle des Kaschemirschafe ist Flaum – den Hasenhaar ähnlich, seiner als das des Elektoralschafes. Mit der klimatischen Modificirung in Spanien und Deutschland, diesem Kaschmirschaf an Körperbau und Wolle am ähnlichsten. Ich glaube daher: daß das Negrettischaf, klimatisch zugebildet in Afrika, das Elektoralschaf in Asie ursprünglich entstanden.

Wenn wir nun zu diesen Gründen der sprechendsten Natur unsere Erfahrungen hinzugeben, und die Sprungresultate aus der Kreuzung beider Rassestämme, mit der Konstanz ihrer Nachkommen dergleichen wollten, wie überzeugend müßte der Ausspruch seyn: daß Negretti und Elektor sehr entfernte und in sich sehr verschieden organisierte Arten und Schafstämme sind, die darum in ihrem gemischten Blut und Nachkommen keine Konstanz vererben können, weil diese das Agregat aller unveränderter Körpertheile und nicht allein der Wolle ist.

Endlich glaubt Hr. Baron v. Bartenstein, daß es räthlicher sey, in Wolle bereits gelungene Varietäten, als fehlerhafte Rassestämme zu paaren, indem die Rassestämme selbst nach meiner Aeußerung, Fehler an sich hätten, und es in allen Fällen die Vernunft gebietet, das Edelste mit dem Edelsten zu vereinen. Hier bin ich mißverstanden. Ich gab nirgend die Rassestämme als absolut fehlerhafte Thiere und nur in Absicht auf Wollproduktion als mit relativen, ökonomisch berechneten Fehlern an. Im Gegentheil sagte ich*)[8] im 4. Grundsatz wörtlich: im Elektoralschof ist die höchste Wollfeinheit, im Negretti die höchste Wollmenge organisch ausgebildet, und im 5. Grundfalz: Elektoralstämme, welche nach dem Prinzip der Wollfeintheit inzüchtlich fortgepflanzt werden, geben endlich in ökonomisch-fehlerhafte Wollschafe über, so wie Negretti inzüchtlich nach dem Princip der Wollmenge gezüchtet, endlich grobe Wollschafe werden. Wirklich erscheinet nach dieser meiner Aeußerung und noch mehr nach dem Ausdruck der Natur, das Elektoralschaf als das höchste Musterbild an Feinheit, das Negrettischaf als das höchste Ideal an Wollmenge in Europa. Das Höchste in diesen Haupteigenschaften, bezeichnet ja aber auch das Vollkommenste, das Edelste. Durch Vereinigung dieses Edelsten, wird ja geradezu allein befolgt, was Baron v. Bartenstein ganz richtig als Grundsatz

---

8 Oekon. Neuigk. S. 691.

aufstellt: das Edelste mit dem Edelstein zu vereinen. Die angeblichen Fehler der Rassestämme sind ja nur ökonomisch relativ nach Geld, aber nicht nach Eigenschaften, nach Feinheit, und Wollmenge berechnet. – Die gegen beide Principien, gegen Oekonomie und Konstanz anstössigen fehlerhaften Thiere sind immer nur die Varietäten, die gegen Konstanz heute gut, in nächster Generation schlechter, zuletzt charakterlos werden, wenn die Urstämme nicht regenerierend einschreiten. Das Unedle ist durch das ganze Thierreich die Varietät, der Bastard; das Edle, die Rasse, das Original. Wer vereinet daher das Edelste mit dem Edelsten; ich, der ich für Wollschäfereien die Rassestämme, oder der, welcher dafür die stets wandelbaren Varietäten paaret? – Der größte Fehler, und keiner straft die Zukunft mehr, ist der Mangel an Konstanz.

Mir erübriget nur noch die gewünschte Antwort auf die Frage zu geben: wie nach meiner Ansicht Varietäten zu konsolidirten, konstanten Rasseschlafen ausgebildet werden könnte?

Diese Frage ist leicht und schwer zu beantworten, je nachdem wir an Natur, an Erfahrung und ueberzeugung, oder an Wünsche, Konvenienz und an das Marktgeschrei der jungen Schafzüchter glauben; denn ein mathematisch auflösbares Problem, ist diese Frage heute noch nicht.

Wenn Konstanz nicht mehr und weniger ist, als der getreue Abdruck des Vaters in Sohn (die unsäglichen seinen Nüanzen ausgenommen), der Mohr von Mohren; wenn diese konstante Fortbildung nur Rasseeigenschaft, Rasse aber klimatisch-genetiste Ausprägung der Organisation, eine organische Umbildung, aller äußern und innern, festen und flüssigen Theile, eine konforme Zubildung der einzelnen Organe zum ganzen Organismus eines Rassethieres ist: was erübrigt den schwachen Menschen gegen das starke Schöpfungsprinzip anders, als ihm in Analogien zu folgen, und seine Winke für seine Zwecke zu benützen? –

a) Eine fortgesetzte Anregung der Natur, gleichviel, ob durch Klima oder äußere Gewalt, bestimmt sie endlich nachzugeben, und die Organisation nach dieser Einwirkung zu modifizieren. Seit Moses die Beschneidung der Juden eingeführt, hat man endlich in einigen Exemplaren beschnittene Judenkinder zur Welt kommen sehen. Der Mohr wird heute noch weiß geboren; aber bereits an einigen Körperstellen nach und nach allmählig schwärzer. Den Berg, den Linnäus sich als das Gebirg der Schöpfung gedacht, fand Pallas in seinen Reisen mit wilden konstanten Originalthieren weniger, und in der Mitte Asiens mehr besetzt, als auf der Linnéschen Schöpfungshöhe. Das Eskurialschlaf aus Kaschemire, so wie das afrikanische Negrettischaf, bildeten sich in Spanien, und ersteres in Sachsen durch Klima und Kultur zu festen modifizierten Rassestämmen aus: langsam weit über ein Menschenalter hinaus, ist dieser Weg, aber nicht ohne Folgen.

b) Wirksamer und schneller ist die Umbildung, so wie die Consolidirung auf dem genetischen Weg der Paarung und Zeugung. Hier werden unmittelbar und schnell zwei Formen in eine verschmolzen.

c) Kultur darf also nur durch wiederholte Zeugung die Natur anregen, in dem Bild des Ideals oder des wirklichen Wahlschafes fort zu erschaffen, und so oft eine Abweichung des Musterstammes eintreten sollte, durch neue Paarungsindividuen die drobende Abweichung auf genetischem Weg, zur Rück- kehr zu zwingen, dadurch, daß man z. B. ein Schaf von zu kurzer feiner Wolle, mit einem Schaf von längerer feinen Wolle und umgekehrt, belegen läßt.

d). Man befolgt dadurch das Gesetz der äußern Anregung für die Consolidirung und das Gesetz der Genesis für die Konstanz zugleich.

Andere Wege gibt es in der Natur und Kunst nicht. So hat Bakewell sein Fleischschaf erschaffen, so hat sich in Deutschland das Elektoratschaf erhalten, und so hat sich das englische Pferd aus der Mischung mit dem Araber fest, konstant gebildet.

Darum müssen wir nach meinem Grundsatz, sub 10, die Urstämme in größter Reinheit erhalten, um mit ihnen, bei Varietäten und Feinwollschäfereien, stets fertig einzufallen, sobald sich Abweichungen, die wir bei unsern Wahlschafen entfernt wünschen, offenbaren. Alles Uebrige ergibt sich, Wiederholung zu vermeiden, aus meinen Grundsätzen von

selbst. Wie bald aber die Natur das Absehen unserer Anregung bewilligt, wie lange und wie oft wir durch genetische Einschreitung die Leiter der Veredlung auf und nieder steigen müssen, wann und ob endlich eine, aus beliebten, für unsere Zwecke und Wollkultur gewählten Varietät resultirende Konstanz, ein unwandelbarer Typus, ein Rassegeschlecht werde, dazu war mein Leben nicht lange genug, um bestimmt eine Zeit oder nur die Zahl der Generationen anzugeben, in denen dieses bewirkt werden könne, meine Erfahrung hat mir darüber immer abweislichen Bescheid gegeben, und so erlaube ich mir auch hier mit fortgesetzten Grundsätzen zu enden, wie ich solches in meiner geschichtlichen Skizze über die österreichische höhere Schafzucht gethan und ersuche dort nach 10 anzureihen, was da folgt:

1 1) Von der reinen Lehre über Rasse, Varietät und Konstanz im Thierreich ist die fortschreitende und stillstehende Veredlung der hochfeinen Schasucht, und die Fortdauer des deutschen Wolls handels abhängig

1 2) Klima, Kultur und Genesis, d. i. Paarung und Zeugung, haben das Geschlecht der Schafe, in Stämme und in Raffen getheilt,

1 3) Genetische Vermischung der Stämme brachen vielerlei Varietäten hervor.

1 4) Rasse ist ein also consolidirter, durch alle Organe so fest ausgeprägter Stamm, der sich unter allen Gegenwirkungen, unter sich konstant fortzupflanzen befähigt ist.

1 5) Varietäten sind das Produkt der Paarung zweier verschiedener Stämme oder Varietäten. Sie haben nicht das innere, feste, organische Ge- präge, daß sie sich konstant fortzupflanzen vermochten, und schlagen, mehr oder weniger auf ihre Urstämme zurück.

1 6) Die Rückschläge geschehen, nach dem Recht des Stärkern unter Mitwirkung des Klimas. In Europa wird der Mulatte weißer, in Afrika schwarzer Mensch. Die Lämmer des Negretti und Elektoralschafstammes, werden im Rückschlag Negretti, weil Negretti fester typirt sind als Elektorals.

1 7) Konstanz ist das durch alle gebildete Gepräge, was sich raffförmig unter allen Bedingungen konstant fortzupflanzen vermag wie der Mohr mit der Mohrinn in Europa.

1 8) Asien war wahrscheinlich die erste, Schöne pfungsstätte der Menschen und unserer Hausthiere. Was damals und dort gelang, werden wir in Europa nicht, noch weniger in Amerika, vollbringen, Unsere besten Hausthiere sind Thiergattungen, asiatischer Kultur unter asiatischem Himmel.

1 9) Aus dieser ursprünglich asiatischen Kultur, bilden sich Stämme, welche akklimatisiert die Natur in ihrer ganzen innern Organisation, und in allen festen und flüssigen Theilen konform umgeändert, und unter dem unbeugsamen Riesenarm der Konstanz zu Rassen in perschiedenen Welttheilen ausgebildet hat.

2 o) Unter europäischer Kultur haben, sich in neuerer Zeit mit etwas temporärer Nachhilfe, das Elektoralschaf in Sachsen und das Fleischschaf Bakewell's in England, rasseförmig gestaltet.

2 1) Schwer geht die Natur in fremde Formen über, noch schwerer gibt sie dieser neuen Form Konstanz und Fortpflanzungsvermögen. Die Macht der Elemente ist an den Kreislauf bestimmter Organisationen gebunden, aus dem Natur selbst schwer weichen kann.

2 2) Das Negrettischaf ist afrikanischen, das Elektoralschaf asiatischen Ursprungs, daher fehr entfernt verwandt und gleichsam das Eine der Mohr, das Andere der Weiße, zu zwei sehr verschiedenen Stämmen gehörig.

2 3) Das Elektoralschaf ist das höchste und edelste an Feinheit, das Negrettischaf, das höchste und vollkommenste an Menge der Wolle. Ihre ökonomisch relativen Fehler entziehen ihnen die organische Vollkommenheit nicht. Wer beide Stämme paaret, vereiniget das Edelste mit den Edelsten. Wer Varietäten paaret, vereinet das Unvollkommene mit dem Unvollkommenen.

2 4) Der größte Fehler an höchst veredelten Schafen ist der Mangel an Konstanz.

2 5) Konstanz bei Varietäten, kann nur langsam errungen werden, und dadurch, daß die Natur unausgesetzt angeregt werde, unser Ideal oder Wahlschaft zu konformieren.
2 6) Der Ausbildungsschritt einer Varietät zur Konstanz ist die durch Paarung und Zengung erfolgende Verschmelzung zweier Wesen in Eines.
2 7) Die wirksamste Nachhilfe leistet man zu dieser Rasseausbildung, wenn jeder Abweichung vom Original mit genetischer Einschreitung dadurch begegnet wird, daß man z. B. zu kurzwollige Mütter mit Widdern von längerer Wolle begattet und umgekehrt.
2 8). Diese Einschreitung wird so lange nöthig, bis die Natur über kurz oder lang aus sich selbst die konstante Fortpflanzung sicher stellt.
2 9) Nur durch Originalstämme können Abweichungen genetisch verbessert, und deßhalb müssen diese in steter Evidenz erhalten werden.
3 0) Binnen welcher Zeit die Natur auch unter dieser Anregung und Nachhülfe die Varietät in Rasse, das Wandelbare konstant ausbilde, kann aus Mangel an Erfahrung nicht und vor der Zeit von Niemand, angegeben werden.
3 1) Was die Wolle betrifft: so sind die Grundsätze zu ihrer gerechten Würdigung weniger im Reinen, als die Lehre über Konstanz. Die Fabrikanten sind allerdings technologische Wollkenner für die Fabrikation; die Wenigsten kennen aber Wolle auf dem Schaf und noch weniger sind die Wollkenner für die Schafzüchtung. Moro ist mir, als Tuchfabrikant berühmt, ein kompetenter und fachkundiger Richter, indem er sagt: keine Wolle sey ihn zu wenn sie nur sein sey.
3 2) Die kurze Wolle ist nicht wegen der Fabrikation, sondern wegen der Züchtung wählbar. Die Natur vollendet im kurzen Haare schneller. Nicht, daß sie ein vorzüglicheres Tuch gäbe, habe ich die Hrn, von Moro aus meiner langen Elektor als wolle schon Anno 1812 ein Tuch fabrizieren seyen, was ich heute umsonst suche. Hat der lange Faden, die Feinheit und Vollendung des Kurzen: so ist er besser als der kurze zu brauchen. Im Gegentheil zu den delikaten Farben, wie Scharlach, wo die Spitzen abgeschnitten werden müssen, taugt keine kurze Wolle.
3 3) Das kurze Haar fiel den Fabrikanten so zufällig in die Hand, wie dem Schafzüchter. Keiner hat die Gründe a priori, alle beide nur die Folgen a posteriori aufgefunden. Der Fabrikation dient kurze Wolle in der Tuchmanufaktur als Bequemlichkeit: dem Schafzüchter schadet das gar zu kurze Haar an Wollmenge. Der Fabrikant sindet das kurze Haar im Faden ausgeglichener und vollendeter, in Spitzen weniger verwittert; er wählt es darum für die Tuchfabrikation lieber. Es schadet feinem Fabrikat jedoch durch weniger schmeichelndes Gefühl und gröberen Griff, obschon es sich ohne die Spitzen zu entfernen, leichter färbt. Der Züchter bemerkt in der kurzhaarigen Zucht und Art die Veredlung schneller, sorgloser zu beschaffen und die Käufer für Wolle und Zuchtvieh modisch leichter anzuziehen. Wenn man den Wollfaden des kurzen und langen Haares gesponnen, vergleichend unter das Mikroskop fetzt: so offenbaren sich im Faden vom kurzen Haar mehr Knöpfe als Vereinigungspunkte der Fäden, die zwar im Tuch, Reißmaschine und Appretur deckt, aber auf Dauer und feinen Griff gegen längere Wolle macht heilig wirkt. Im Tuch sind die kurzen Wollfehler leichter zu verstecken; zu edleren Fabrikaten, zu feinfarbigen Tüchern, zu Scharlach, zu Shawls, zu hochfeinen Merinos und Zeugen, zu allen Tüchern, die Leichtigkeit mit Dauer verbinden sollen, taugen kurze Wollen nicht oder weniger. – Das und Mehr, ist Hrn. Elsner auf die Wollart zu antworten, die er in Bastard wollen als die heute Vorzüglichste rühmt und als ein eben so ephemeres Produkt in der Fabrikation, wie das kurzwollige Schaf in der Züchtung sich darstellen wird. In der Mitte liegt die Wahrheit.
Es ist eine bekannte Sache, daß durch die Schafwolle mehr Geld aus dem Ausland einfließt, als durch die Ausfuhr aller übrigen Produkte. Ob eine solche ernste Landesfache, die erste des Aktivhandels, nicht eine Staatsanstalt, eine Ermunterung, eine Unterstützung verdiene, da sich alles um uns beeilet und bestrebt, Oesterreich das goldene Vließ zu entwinden, überlasse ich den Patrioten und den Wenigen, die die Wichtigkeit davon zu würdigen vermögen oder belieben. Oesterreich hat das Eis gebrochen, mußte um eine Infassen aufzuklären, zur Publicität greifen und indem sie

ihm, wie hier, die Resultate älterer Erfahrung mit heilt, das Ausland mitaufklären. Es gibt aber dennoch eine Wissenschaft außer der bereits bekannten, die man lange bei großen Anstalten benützen, und die uns den Vorzug lange, lange sichern kann.

J. M. Freiherr v. Ehrenfels.

2.

Notices
of the Imperial and Royal Moravian-Silesian Society for the Promotion of Agriculture,
of natural and regional history in Brno.

Nro. 18.

May 1831.

Continuation of the thoughts of Mr. Moritz Beyer about the Merino sheep
By J. M. Freiherr von Ehrenfels.

Mr. M. Beyer, with his ideas about the Merino sheep, has taken a deeper look into nature than many of his predecessors. However, as certain as it is that the sheep family appears in many different forms on our earth, it is one and the same species everywhere; the Merino and Electoral sheep cannot be called the creature of human industry.

Nature allows three potencies to act favourably or unfavourably on animal organizations. They are: climate, nourishment, and procreation. Intelligence and culture - human industry in general - are not subject to the above three principal forces, and can only act through them.

Procreation, the union of two beings, gives the living form to the dead chaos of matter. This genetic force, the beginning of life and of the formation of dead matter, can hardly, and gradually only through climate and nourishment, mesh with its gears. An immutable law governs the shaping of forms; it is not the wishes and desires of men that rule; nature herself obeys her institutions.

If we could see the miracle of dead matter coming to life, how we would be amazed! A living point is created out of the globules between which the juices are squeezed, and out of this point a creature of the earth is born. Even genetic power cannot act arbitrarily here; it must follow the type that has been formed in advance and inwardly to life and form.

What Harvey, what Wolf and others handed down *de generatione animali*, deserved a popular translation for the animal breeder. Only climate, nourishment, and procreation created the various divisions of this or that species of animal. In natural history, we erroneously call these divisions breeds or hybrids. The word, however, refers to the difference in descent, the word hybrid to deviating or failing reproduction; varieties of the genus are species.

I would rather call the Merino sheep a creature of chance than of human industry. The evidence that Bakewell gave his animals the imprint of his will, and in Spain, as with us, other sheep varieties besides Merinos also exist, decides it for me.

Bakewell did not find his meat sheep in a chemical laboratory, or form it by dissolution and precipitation of matter; he merely obtained it by procreation and nourishment insofar as his climate cooperated kindly or hostilely. - –

He placed the first sheep with the rounded, barrel-shaped, full-fleshed figure on his fattening fields, planted it among itself, did not disturb the procreation by opposing forms, created for it above all a consolidated nursery, and obtained a variety of sheep such as climate, procreation, and diet allowed to arise.

That in Spain, besides the fine-wooled wandering sheep, under the same climate the coarse-wooled standing sheep, as in Saxony, next to each other II. Issue 1831. persists, gives proof that climate and industry alone cannot create and maintain a Merino sheep, and only climate, diet and procreation together can.

Because horse and donkey coexist in one country and climate, the horse cannot be called a creature of industry because the donkey has not already become a horse climatically.

We do not know how some of our present species of animals, in an earlier state of the earth, in a luxuriantly nourishing climate, under the irritable solar warmth of Asia, under the first electric outpouring of the life-force for the further development of procreation and the sexual instinct, might have been able to mate with one another; but this we see, that in our present developed, even antiquate world, the boundaries of reproduction are genetically separated, and in the state of nature no wild animal intermarries any longer with an alien species. Reproduction has narrowed down and limited itself to the rule of the species. Even the horny ibex seeks only his equivalent.

Human industry presupposes, as it were, a human driving wheel, which outwardly forms, separates and joins, smoothes or stacks the wool, spins a coarse thread into a fine one. That we do not have this machinery, and may at most bring Genesis into position to shape it more according to human purposes, is all we can do; and this endeavour, whereby the breeder guides nature not with things from without, but with intellectual stimulation from within, I would rather call natural wisdom than human industry. The above three powers: climate, nourishment, and procreation, remain the levers of nature and of the formation of matter.

Here, too, experience reaches out to us. - We do not know historically how the Escurial sheep formed from the Spanish divisions of the pure-bred sheep, but we do know historically exactly how the Electoral sheep and even some of its varieties were formed. From the already firmly typified Escurial strain and, according to the principle of fineness, also selected from other herds, a number came to Saxony. Here they were moderately nourished, reproduced among themselves, and left indifferent to the German climate. Without any industry this was what they could become under the above three powers in Lohmen.

For the fact that the Electoral sheep therefore did not come from Spain as a refining agent, as we now hold, is a settled truth. If even the iron-shod measuring rod shortens in hard frost, how should the force of the climate not act on animal fibres and skin organs? - If Rochsburg, like Bakewell in England, had soon won its own division of the Electoral sheep by stable and fattening feed, how should not, with scarcity and abundance, modifications appear here and there and everywhere? - If one understands nature, and lets better principles of mating and procreation prevail, how should not the constancy finally develop and the type become more uniform in continued generations? - Not as Canova can arbitrarily create a Venus or a cobblestone from a lump, can the animal breeder dispose of the genetic organic power.

I only wish to establish the proposition for the sake of inference: that human industry is not absolute, least of all for itself, but only through the somewhat guided force of nature. The granting of this principal power to our desires will not depend on human industry, but, whether successful or unsuccessful, it will depend on the interaction of the three powers, of which procreation, as the genetic power, is the most powerful.

According to these premises, the Electoral sheep, like the Merino sheep in general, is not a creature of human will or of an industry, but the result of a constantly progressing metamorphosis of the sheep and all animal species on earth, just as the interaction of the powers governing the form caused them to result. The animal species that could not endure this metamorphosis perished with the old world.

I cannot follow all of Mr. Beyer's intellectual ideas to this extent. Some of them stand purely for themselves, and would not succeed if one wanted to penetrate deeper into their core. I would like to take the liberty of making a few additions from the book of my experience only about the formation of the Electoral sheep as it developed with regard

to wool because at the same time they contain a modest correction by the writer and collector of everything worth knowing about sheep breeding, Mr. Wirthschaftsrath B. Petri, who is held in high esteem by me.

When the original strain of the Electoral sheep came from Spain, it was in the Escurial sheep less in wool than a German descendant, but in the conflict of several good qualities less with the defects of today's offspring, i.e., more perfectly handed down. Climate, diet and procreation developed together what nature, for the time being in Lohmen, could work out. It was a consequence of genesis and mating, as soon as the ram was chosen solely on the principle of wool fineness, that at Lohmen the yarn of the sheep first manifested itself with an incredible wool reduction. With meagre food and pasture, where the wool hair lengthened less than elsewhere, one noticed this defect earlier in the wool weight than with the eye, and so this defect became almost constant.

This was noticed earlier at Rochsburg with fattening and stable fodder. Here the bodies enlarged unbelievably and caused the skin to expand accordingly. The skin organs or hair follicles cattered there that were not preformed by breeding from within and the skin expanded accordingly; had the hair bulbs in the extended skin increased according to the expansion of the skin, but were only more developed and moved apart by means from the outside, with an already innate texture of the skin, the strongest nourishment could just as little create more hair bulbs in the extended skin as more than two eyes on the head.

Each wool hair that sprouts requires a bulb in advance, where this is missing, no hair grows and the skin remains bare. The larger, therefore, the body, the more stretched the skin, the more stretched this, the farther apart the hair bulbs, the thinner-wooled the sheep. - The hair bulbs in this less crowded position enlarged, perfected themselves, like the aerated tree in thinned forests, and according to this proportion supplied the wool hair with several nutritive juices.The increased food could not be consumed in any other way than by lengthening the hair itself, as the hair from their basic organs could not multiply, and so a division of the Electoral sheep arose in Rochsburg, which had wool from 4 to 5 inches long and replaced the weight with its length of thread for the time being.

This kind of sheep was criticized in our time, and regressed through breeding. - Not everyone knew what they were doing. This wool product, both in terms of monetary results and in terms of factory use, withstands all competition, and, for the present, was the only way to improve the innate defect of the Electoral sheep. The longest, finest worsted wool was obtained there, and it could also be used for cloth. The weight increased genetically more and more, and what this variety of sheep may achieve in totality, I have shown in the system of my new sheep culture: the Electoral sheep is of the type to use two and three shearings a year.

The rational sheep breeder Mr. Schnetger of Machern operated differently. He was looking for short, stout, fine wool, and comparatively soon found that he was breeding a different kind of sheep compared to Rochsburg. He had smaller livestock, and therefore with less extensive skin a denser stand of the wool hair by itself. He fed moderately, and did not cause any artificial influx of the wool-lengthening juices. He looked for sheep with an originally suitable skin formation, and worked through Genesis on the constancy of his thus formed Electoral sheep. Thus, within a few hours of each other, in Saxony itself, we experienced two divisions of the Electoral sheep, which in some wool characteristics are opposed to each other, - all through the main force of procreation, in conflict with climate and diet.

Mr. Wirthschaftsrath Petri, who in these pages (Nro. 48 v. J.) invited attempts to make the sheep’s wool denser by frequent shearing in the first years of life, may, after the foregoing hints, wish to modify his theory. Frequent shearing, as a means from the outside, remains without effect for the skin organs and multiplication of the hair bulbs on the inside. One cannot make a denser fleece in any other way than by multiplication of the hair bulbs from the inside out, and this is possible only through breeding.

Nature controls new ideas most exactly. Let us also assume that in young lambs, as with young, suppressed trees in the dense forest, suppressed bulbs of the skin gain space and development through breathing room and that which is caused by the prepotency of the stronger ones,
previously suppressed, and could develop somewhat more finely: so the skin organ, in which the hair bulbs with their own nutritive vessels like an isolated body standing there must at the same time be provided from the inside with vessels leading to and from them, without which the hair bulb would remain a dead point. Can this be thought possible from the outside through the scissors? And does such a presupposition not obviously contradict the economy of nature?

I conclude, like Mr. Beyer, with the remark that the Electoral sheep is not the highest ideal of higher sheep breeding. Nature has allowed us too deep a view into her workshop for us to be able to dismiss her without being asked to deliver her best. - No one has yet dared to express this ideal in definite terms. I describe it very simply: it is the Escurial sheep that was already there and has been lost, -a sheep which combined the fineness of wool of the Electorals with the quantity of wool of the Negretti. Nature had already worked it out in the Escurial, otherwise the Electoral sheep, not a specimen formed upwards as a whole but individually downwards, could not have resulted from it under German skies. The restoration of this type of sheep is possible because it was already there. In what way, despite all the hints, remains the secret of breeding and the property of a trained, watchful eye, familiar with nature and creation. It will be difficult to find a German Bakewell, because this creation requires the progressive, persevering common spirit of a corporation instead of the finite spirit of the individual and presupposes many combined forces and participation.

It cannot be called The German Merino sheep, because it is still uncertain who will first and most happily enter the secret workshop of nature, which country will first make this conquest, and where the new Jason will plant the golden fleece, in Europe or America, in Germany, France, or Russia. - It must be called the restored Escurial sheep, -a name which remains applicable to all countries, and to the summary property of the subject alone. Spain, with the introduction of true Electoral sheep, born constant in strict inbreeding, already in possession of pure Negretti, would have the first claim climatically. Climatically, Hungary comes next.

Germany is in great danger, its intelligence notwithstanding, of departing more and more from this aim and ideal. It believed that it already possessed the German Merino sheep in its own inconstant wool sheep, resulting from the ordinary arbitrary mixture of the Electorals and the Negretti, or even from the composition of their varieties. Against this, I wrote my treatise on *Rasse, Varietät und Konstanz*, which I here ask you to read for completeness (*Mittheilungen* No. 17 and 18 of 1829). - The Escurial sheep has unfortunately been lost in Spain and Germany; only remnants still live in the Electoral sheep. The pure, constant, inbred consolidated Electoral sheep in the urge to improve its faults is seldom to be found; the Negretti strain is also quite changed according to the demand of the day. Where shall we ultimately seek the elements from which the Escurial sheep can rise again? - –

If Germany is to reach the goal set, it can only be done by first seeking, cultivating and purifying the Electoral and Negretti strains of pure, inbred descent, and then itself developing them as a pure nursery from which the ideal is realized through rational interaction, and, indeed, until the consolidation of this new Escurial pure-bred strain, always refreshed from the above sources, used and perpetuated at every setback, receiving new genetic power for the upswing of highest constancy.

The noble wool of Germany, as it is now, will soon be imitated in other countries. The excellence of wool, on which the most important, indeed the first, branch of the German foreign trade and its guarantees depend, is based on the authenticity of the sheep strain. This category deserves a state institution; for wool brings in more money than all the other export articles put together. - But will we only establish it when it is perhaps too late and Germany has already been surpassed in other countries? Also, the elements for regeneration may already be too spoiled, and the will only be

found together with the regret of not having had such an institution earlier? Have the experiences that could bring the Escurial sheep back to life perhaps not already perished at the same time as the learned practitioners of our time?

I had suggested that this somewhat academic institution be established privately in the fortunate land of the Magyars. It is not in my nature to use other insinuations than the publicly expressed truth and disinterestedness to achieve this. Notwithstanding the enthusiastic participation of many, the venture, based on shares, seems to be delayed and thus to fail. Perhaps the idea is rising in a more limited circle elsewhere. How easy it would be for a few friends to establish such an institute, depositing their experiences everlastingly as if in a temple of Isis and Osiris! Who could doubt that they built, according to this spirit, with the highest interests for an eternity? - –

Mr. Beyer may take it that what I have just presented is not a critique of his thoughts, but a continuation of his ideas. Mr. Beyer's essay, which prompted this, in its polite, moderate, modest outline, deserves, instead of criticism, the most loving encouragement to continue his ideas and researches. Whoever creates a lie out of nature and a truth directly or indirectly in our midst, is more a hero of his time than those in the old world who killed a monster. We need not demoralize ourselves or become malicious at the friendly communication of modified ideas. By a modest opposition the truth grinds itself purer; only the personality, in the mask of this, remains deadly and ugly. - The economist does not always write for an ungrateful crowd. Even though this kind of writing is often confused with poor intellectual products, and seldom rewarded with more than a pitying, mocking acquiescence: yet spiritual, humane ideas always remain guarantors of those who will be more intellectual. Forward, like nature, goes the banner of the thinker and the rational economist. - –

Enlightening the field of agriculture is the most humane work of the time, to which the better ones are all kindly invited. As the world is shaping up today, thousands of educated people must swear by the plough, or starve, or take the walking stick to the wastelands of America and Russia, where they again find only the plough as the aegis of life. As once the Persian worshipped the sun as his nearest benefactress, so in Europe thousands from other too fully refined classes must soon worship the plough. In Germany the father of a family already regards it as a good provision for his son; when he serves as a common soldier, an otherwise so avoided obligation, is accommodated, and what does the sad emigrant seek but a bit of earth, which he is denied in his fatherland? The German earth is becoming too narrow because we are building it only with the brute strength of the common farmer; for rational farmers it still holds room for a hundred million people.

Mittheilungen
der k.k.Mährisch-Schlesischen Gesellschaft zur Beförderung des Ackerbaues, der Natur- und Landeskunde in Brünn.

Nro. 18.

Mai 1831.

Fortsetzung der Gedanken des Herrn Moritz Beyer über das Merinoschaf
Von J. M. Freiherrn von Ehrenfels.

Herr M. Beyer hat mit seinen Ideen über das Merinoschaf einen tiefern Blick in die Natur gethan, als viele seiner Vorgänger. So gewiß es jedoch ist, daß in fo verschiedenen Formen auch das Schafgeschlecht auf unserer Erde erscheint, es doch überall eine und dieselbe Gattung sey; so wenig kann das Merino- und Elektoralschaf mit das Geschöpf menschlicher Industrie genannt werden. Drei Potenzen läßt die Natur auf thierische Organisationen günstig oder ungünstig einwirken. Sie find: Klima, Nahrung und Zeugung. Intelligenz und Kultur – menschliche Industrie überhaupt – sind den obigen drei Prinzipalkrästen unthan, und können nur durch sie wirken.

Zeugung, die Vereinigung zweier Wesen, gibt dem todten Chaos der Materie die lebendige Form. Diese genetische Kraft, der Anfang des Lebens und der Formation todter Materie, läßt sich kaum, und allmählig nur durch Klima und Nahrung in ihre Räder fallen. Ein unwandelbares Gesetz waltet in Gestaltung der Formen, nicht die Wünsche und Gesuche der Menschen regieren, die Natur selbst gehorcht ihren Institutionen.

Könnten wir das Wunder sehen, wie die todte Materie ins Leben tritt, wie würden wir staunen! Aus Kügelchen, zwischen welche Säfte schietzen, wird ein lebendiger Punkt, und aus diesem Punkt erzeugt sich ein Geschöpf der Erde. Selbst die genetische Kraft kann hier nicht willkührlich schalten, sie muß dem Typus, der sich da zum Leben und zur Form voraus und innerlich gebildet, folgen.

Was Harvey, was Wolf u. A. de generatione animali überliefert, verdiente für den Thierzüchter eine populäre Uebersetzung. Nur Klima, Nahrung und Zeugung zusammen hatten die verschiedenen Abtheilungen dieser oder jener Thiergattung gebildet. Naturhistorisch nennen wir diese Abtheilungen irrthümlich Raffen oder Bastarde. Das Wort Raffe leitet jedoch auf Verschiedenheit der Abstammung, das Wort Bastard weitet auf abweichende oder versagende Fortpflanzung; Varietäten des Genus sind die Species. Eher möchte ich das Merinoschaf ein Geschöpf des Zufalles, als der menschlichen Industrie nennen. Die Belege, daß Bakewell seinen Thieren, das Gepräge seines Willens aufgedrückt, und in Spanien, wie bei uns auch andere Schafvarietäten neben Merinos existieren, wenden sich für mich. Bakewell hat sein Fleischschaf nicht in einem chemischen Laboratorium gefunden, oder durch Auflösung und Niederschlag der Materie geformt; er hat es bloß durch Zeugung und Nahrung in so weit erlangt, als sein Klima freundlich oder feindlich mitwirkte. –

Das erste Schaf mit der abgerundeten tonnenförmigen, vollen Fleischfigur setzte er auf seine Mastfelder, pflanzte es unter sich fort, störte die Zeugung nicht durch entgegengesetzte Formen, schuf sich vor Allem eine konsolidierte Pflanzschule, und erhielt eine Schafvarietät, wie es Klima, Zeugung und Nahrung auferstehen ließen. Daß in Spanien neben dem feinwolligen Wanderschaf, unter demselben Klima das grobwollige Standschaf, wie in Sachsen, neben einander II. Heft 1831. fortbesteht, gibt den Beweis, daß Klima und Industrie allein kein Merinoschaf, und nur Klima,

Nahrung und Zeugung zusammen dieses schaffen und erhalten können. Man kann weil Pferd und Esel in einem Lande und Klima zusammen fortbestehen, das Pferd deßwegen kein Geschöpf der Industrie nennen, weil der Esel nicht schon klimatisch zum Pferd geworden.

Wir wissen nicht, wie manche unserer jetzigen Thiergattungen in einem frühern Zustande der Erde, in einem üppig nährenden Klima, unter der reizbaren Sonnenwärme Asiens, unter der ersten elektrischen Ausströmung der Lebenskraft zur Fortbildung der Zeugung und des Geschlechtstriebes an einander gegangen, oder sich gepaart haben; aber das sehen wir, daß bei unserer heutigen ausgebildeten , ja veralteten Welt die Grenzen der Fortpflanzung geuetisch geschieden sind, und im Naturstande sich kein wildes Thier mehr mit einer fremden Gattung vermählt. Eingeengt hat sich die Fortpflanzung und auf die Regel der Gattung beschränkt. Selbst der geile Steinbock fucht nur seines Gleichen.

Menschliche Industrie fetzt gleichsam ein menschliches Triebrad voraus, was äußerlich Formen bildet, trennt und verbindet, die Wolle glättet oder stapelt, aus dem groben Faden einen seinern spinnt. Daß wir diese Maschinerie nicht haben und höchstens die Genesis in Lagen bringen mögen, mehr nach menschlichen Zwecken zu formen, das ist Alles, was wir können; und dieses Bestreben, wobei der Züchter die Natur nicht mit Dingen von außen, fondern mit geistiger Anregung von innen leitet, möchte ich eher Naturweisheit, als menschliche Industrie nennen. Obige drei Potenzen: Klima, Nahrung und Zeugung, bleiben die Hebel der Natur und der Formation der Materie.

Auch hier reicht uns Erfahrung die Hände. – Wir wissen geschichtlich nicht, wie sich das Eskurialschaf aus den spanischen Abtheilungen des Edelschafes gebildet habe, aber wir wissen geschichtlich genau, wie sich das Elektoralschaf und selbst einige seiner Varietäten gemach haben. Von dem bereits fest typirten Eskurialstamm und, nach dem Prinzip der Feinheit, auch aus andern Heerden gewählt, kam eine Anzahl nach Sachsen. Hier wurden sie mäßig genährt, unter sich fortgepflanzt und dem Ohne alle Industrie ward, was unter obigen drei. Potenzen in Lohmen werden konnte.

Denn daß das Elektoralschaf nicht also aus Spanien kam, wie wir es als Veredlungsmittel heute besitzen, ist eine abgemachte Wahrheit. Wenn sich selbst die mit Eisen beschlagene Meßstange bei hartem Frost verkürzt, wie soll die Gewalt des Klima nicht auf animalische Fibern und Hautorgane wirken? – Wenn Rochsburg durch Stall- und Mastfutter, wie Bakewell in England, eine eigene Abtheilung des Elektoralschafes bald gewonnen hatte, wie sollten nicht, bei Mangel und Ueberfluß, dort und da und überall Modifikationen erscheinen? – Wenn man die Natur versteht, und nach bessern Prinzipien Paarung und Zeugung walten läßt, wie sollte nicht die Konstanz endlich stegen und der Typus in fortgesetzten Generationen gleichartiger werden ? – Nicht wie Canova aus einem Klumpen eine Venus oder einen Pflasterstein willkührlich erstehen lassen, kann der Thierzüchter über die genetiche organische Kraft verfügen.

Nur wünsche ich den Satz, um der Folgerung willen, aufzustellen: daß menschliche Industrie nicht absolut, am wenigsten für sich, sondern nur durch die etwas geleitete Kraft der Natur wirken möge. Welche Gewährung diese Prinzipalkraft unsern Wünschen gestattet, wird nicht von menschlicher Industrie, wohl aber, gelungen oder mißlungen, von der Wechselwirkung der drei Potenzen abhängig bleiben, unter denen Zeugung, als die genetische Kraft, die mächtigste da steht.

Nach diesen Prämissen bleibt das Elektoralschaf, so wie das Merinoschaf überhaupt kein Geschöpf menschlichen Willens oder einer Industrie, sondern das Resultat einer beständig fortschreitens den Metamorphose des Schaf- und aller Thiergeschlechter auf Erden, so wie unter Zusammenwirkung der die Form regierender Potenzen es diese entfallen lassen. Thiergattungen, welche diese Metamorphose nicht aushalten konnten, sind mit der alten Welt untergegangen.
Ich kann nicht allen geistigen Ideen des Hrn. Beyer in dieser Ausdehnung folgen. Manche stehen rein für sich selbst da, und würden nicht gewinnen, wollte man tiefer auf ihren Kern dringen. Nur über die Bildung des Elektoralschafes wie es sich in Absicht auf Wolle gestaltete, möchte ich mir einige Zusätze aus dem Buche meiner Erfahrung erlauben, weil

sie zugleich eine bescheidene Berichtigung des mir schätzbaren Schriftstellers und Sammlers alles Wissenswerthen über Schafzucht, des Hrn. Wirthschaftsraths B. Petri, mit enthalten.

Als der Urstamm des Elektoralschafes aus Spanien kam, ward er im Eskurialschaf zwar minder an Wolle sein, als eine deutschen Nachkommen, doch im Conflikt mehrerer guter Eigenschaften weniger mit den Fehlern der heutigen Nachzucht, d. i. vollkommener überliefert. Klima, Nahrung und Zeugung entwickelten zusammen, was die Natur, vorerst in Lohmen, ausarbeiten konnte. Es war eine Folge der Genesis und Paarung, sobald man den Sprungbock bloß nach dem Prinzip der Wollfeinheit wählte, daß sich zu Lohmen das Zwirnen der Schafe mit einer unglaublichen Wollverminderung zuerst offenbarte. Bei karger Nahrung und Weide, bei welchen sich das Wollhaar da weniger, als anders wo verlängerte, bemerkte man diesen Fehler früher im Wollgewicht, als mit dem Auge, und so wurde dieser Fehler beinahe konstant.

Früher bemerkt wurde dieses zu Rochsburg bei Mast- und Stallfutter. Hier vergrößerten sich die Körper unglaublich und veranlaßten eine darnach bemessene Ausdehnung der Haut. Die Hautorgane oder dort zerstreuten Haarzwiebeln, die da nicht durch Zeugung von innen präformiert und der Hautausdehnung gemäß vermehrt, fondern durch Mittel von außen nur mehr entwickelt und auseinander gerückt waren, konnten bei bereits angeborner Textur der Haut durch die stärkste Nahrung eben so wenig mehr Haarzwiebeln in die ausgedehnte Haut als am Kopfe mehr wie zwei Augen schaffen.

Jedes Wollhaar sproßt und fetzt eine Zwiebel voraus, wo diese fehlt, wächst kein Haar und die Haut bleibt nackt. Je größer daher der Körper, desto gedehnter die Haut, je gedehnter diese, desto weiter auseinander die Haarzwiebeln, desto dünnwolliger das Schaf. – Die Haarzwiebeln in dieser weniger gedrängten Stellung vergrößerten, vervollkommneten sich, wie der gelüftete Baum in gelichteten Wäldern und führten nach dieser Proportion dem Wollhaar mehrere Nahrungssäfte zu.

Die mehr zugeflossene Nahrung konnte nicht anders als durch Verlängerung des Haares selbst verbraucht werden, da sie die Haare aus Abgang ihrer Grundorgane nicht multiplizieren konnte, und so entstand in Rochsburg eine Abtheilung des Elektoralschafes, welche Wolle von 4 bis 5 Zoll Längetrug und dem Abgang des Gewichts vorerst durch seine Fadenlänge ersetzte.

Diese Art Schafe wurde zu unserer Zeit getadelt und durch Zeugung zurückgebildet. – Nicht Alle wußten, was sie thaten. Dieses Wollprodukt, sowohl nach Geldresultaten, als Fabriksanwendung, halt jede Konkurrenz aus, und war vorläufig das einzige Mittel, den angeborenen Fehler des Elektroralschafes zu verbessern. Die längste, feinste Kammwolle ward da gewonnen, und auch zu Tuch blieb sie brauchbar. Das Gewicht vermehrte sich genetisch mehr und mehr, und was diese Schafvarietät der Totalität nach leisten mag, habe ich im System meiner neuen Schafkultur, das Elektoralschaf der Art, zwei- und dreischürig zu benutzen, dargethan.

Anders manipulierte der rationelle Schafzüchter Herr Schnetger auf Machern. Er suchte kurze, gedrungene, seine Wolle, und fand vergleichend bald, daß er gegen Rochsburg eine andere Art Schafe züchtete. Er hatte kleineres Vieh, und daher bei minder ausgedehnter Haut dichtern Stand des Wollhaares von selbst. Er fütterte mäßig, und veranlaßte keinen künstlichen Zudrang der die Wolle verlängernden Säfte. Er suchte sich Schafe von ursprünglich zufagender Hautbildung, und wirkte durch Genesis auf die Konstanz feines also gebildeten Elektoralschafes. So hatten wir, wenige Stunden von einander, in Sachsen selbst, zwei Abtheilungen des Elektoralschafes erfahren, die sich in mancher Wolleigenschaft einander gegenüber stehen, - Alles durch die Hauptkraft Zeugung, im Conflikt mit Klima und Nahrung.

Herr Wirthschaftsrath Petri, der in diesen Blättern (Nro. 48 v. J.) zu Versuchen eingeladen hat, das Schaf durch öfteres Scheeren in den ersten Lebensjahren dichtwolliger zu machen, beliebe nach vorstehenden Andeutungen seine Theorie zu modifizieren. Das öftere Scheeren, als Mittel von außen, bleibt für die Hautorgane, und Multiplikation der

Haarzwiebeln, im Innern, ohne Wirkung. Man kann kein dichteres Vließ anders, als von innen heraus durch Multiplikation der Haarzwiebeln erzielen, und dieses wird nur durch Zeugung möglich.

Die Natur kontrollirt neue Ideen am richtigsten. Wollen wir auch annehmen, daß bei jungen Lämmern , wie im dichten Wald junge, unterdrückte Bäume, unterdrückte Zwiebeln der Haut durch Lüftung Platz und Entwickelung gewönnen, und das, was durch die Präpotenz der stärkern, früher unterdrückt, und etwas seiner sich ausbilden könne: so müßte doch das Hautorgan, in dem die Haarzwiebeln mit ihren eigenen Nährgefaßen wie ein isolierter Körper da stehend gedacht werden müssen, mit zu- und zurückführenden Gefäßen, ohne denen die Haarzwiebel ein todter Punkt bleibt, zugleich von innen aus versehen werden. Kann das durch die Scheere von außen wohl möglich gedacht werden? und widerspricht eine solche Voraussetzung nicht offenbar der Oekonomie der Natur?

Ich schließe, wie Herr Beyer, mit der Aeußerung: daß das Elektoralschaf nicht das höchste Ideal höherer Schafzucht sey. Die Natur hat uns einen zu tiefen Blick in ihre Werkstätte gestattet, als daß wir sie unaufgefordert, ihr Höchstes auszuliefern, entlassen könnten. – Es hat noch Niemand gewagt, dieses Ideal bestimmt auszusprechen. Ich bezeichne es sehr einfach: Es ist das bereits da gewesene, und verloren gegangene Eskurialschaf, – ein Schaf, welches die Wollfeinheit der Elektorals mit der Wollmenge der Negretti verband. Die Natur hatte es bereits im Eskurial ausgearbeitet, sonst hätte unter deutschem Himmel nicht daraus das Elektoralfhaf, kein im Ganzen hinaufgebildetes, sondern individuell herabgebildetes Eremplar entfallen können. Möglich wird die Wiederherstellung dieser Schafart, weil sie bereits da war. Auf welchem Wege bleibt bei aller Andeutung doch immer noch das Geheimniß der Züchtung und das Eigenthum eines eingeübten, wachsamen, mit der Natur und der Schöpfung vertrauten Auges. Schwer wird sich ein deutscher Bakewell finden, weil diese Creation. Statt des endlichen Geistes des Einzelnen den fortschreitenden, ausdauernden Gemeingeist einer Korporation erheischt und viele zusammengesetzte Kräfte und Theilnahme voraussetzt.

Das deutsche Merinoschaf kann es nicht heißen, weil noch ungewiß ist, wer zuerst und am glücklichsten in die geheime Werkstätte der Natur eindringt, welches Land zuerst diese Eroberung macht und wo der neue Jason das goldene Vließ aufpflanzt, in Europa oder Amerika, in Deutschland, Frankreich oder Rußland. – Es muß das restaurierte Eskurialfchaf heißen, – ein Name, der für alle Länder und für die summarische Eigenschaft des Gegenstandes allein anwendbar bleibt. Spanien hätte, mit der Einführung wahrer Elektoralfchafe, in strenger Inzucht konstant geboren, bereits im Besitz reiner Negretti, den ersten Anspruch klimatisch. Ungarn kömmt klimatisch am nächsten.

Deutschland steht in großer Gefahr, seiner Intelligenz ungeachtet, sich immer mehr und mehr von diesem Ziel und Ideal zu entfernen. Es glaubte, in dem seinen, inkonstanten Wollschaf, aus der gemeinen willkürlichen Mischung der Elektorals und der Negretti, oder gar aus der Komposition ihrer Varietäten entfallen, das deutsche Merinoschaf schon zu besitzen. Ich schrieb dagegen meine Abhandlung über Raffe, Varietät und Konstanz, die ich hier zur Vervollständigung (Mittheilungen Nr. 17 u. 18 von 1829) nachzulesen erfuche. – Das Eskurialschaf ist leider in Spanien und Deutschland verloren gegangen; nur im Elektoralschaf leben noch Reste. Das reine, konstante, inzüchtlich konsolidierte Elektoralschaf in dem Drange seine Fehler zu bessern, ist selten zu finden; auch der Negrettistamm ist durchaus nach der Tagesforderung verändert. Wo sollen wir am Ende die Elemente suchen, aus denen das Eskurialschaf wieder auferstehen kann ? –

Soll Deutschland zu dem vorgesteckten Ziele gelangen, so kann es nur dadurch geschehen, daß der Elektoral-, wie der Negrettistamm vorerst aus reiner, inzüchtlicher Abkunft gesucht, gepflegt, geläutert, dann selbst als reine Pflanzschule aus der mit rationeller Wechselwirkung das Ideal verwirklicht entfalle, gewissenhaft verwahrt, ja bis zur Consolidirung dieses neuen Eskurial-Edelstammes, immer aus obigen Quellen aufgefrischt, bei jedem Rücktritt, neue genetische Kraft zum Aufschwung höchster Konstanz empfangend, benützt und verewigt werde.

Die Edelwolle Deutschlands, wie sie jetzt ist, wird bald in andern Ländern nachgemacht seyn. Von der Originalität des Schafstammes ist die Vorzüglichkeit der Wolle, von diefen bisherigen Vorzug der bedeutendste, ja erste Zweig des deutschen Aktivhandels und seine Garantien abhängig. Eine Staatsanstalt verdiente diese Rubrik; denn die Wolle bringt mehr Geld ein, als alle übrigen Ausfuhrartikel zusammen. – Doch wir werden sie erst dann etablieren, wenn es vielleicht zu spät und Deutschland bereits in andern Ländern übertroffen seyn dürfte? Auch dürften zur Regeneration die Elemente dann schon zu verdorben, und der Wille sich mit dem Bedauern erst einfinden, eine solche Anstalt früher nicht schon zu haben? Werden die Erfahrungen, welche das Eskurialfchaf ins Leben fördern könnten, vielleicht nicht schon mit den gebildeten Praktikern unserer Zeit zugleich untergegangen seyn?

Ich hatte angeregt, diese etwas geistige Anstalt privatim in dem glücklichen Lande der Magyaren zu etablieren. Es liegt nicht in meiner Art, andere Infinuationen, als die öffentlich ausgesprochene Wahrheit und Uneigennützigkeit zur Erzielung anzuwenden. Der enthusiastischen Theilnahme Vieler ungeachtet scheint die Unternehmung, auf Aktien basiert, sich zu verzögern und dadurch zu scheitern. Vielleicht steht die Idee in einem beschränktern Kreise anderswo auf. Wie leicht könnten einaige Freunde, die ihre Erfahrungen wie in einem Tempel der Isis und Osiris unvergänglich deponirten, ein solches Institut begründen! Daß sie, nach diesem Geist, mit den höchsten Interessen für eine Ewigkeit bauten, wer könnte daran zweifeln? –

Herr Beyer beliebe zu entnehmen, daß ich das so eben Vorgetragene nicht Kritik feiner Gedanken, sondern Fortsetzung seiner Ideen überschrieben habe. Der Aufsatz des Herrn Beyer, der diesen veranlaßte, in dem fo artigen, gemäßigten, bescheidenen Umriß, verdient. Statt Kritik die liebreichste Ermunterung zur Fortsetzung seiner Ideen und Forschungen. Wer eine Lüge aus der Natur und mittel- oder unmittelbar eine Wahrheit in unsere Mitte, schafft, ist mehr Heros seiner Zeit, als in alter Welt jene, die ein Ungeheuer getödtet haben. Wir müssen uns nicht selbst entmuthigen oder böswillig werden über die freundliche Mittheilung modifizierter Ideen. Durch eine bescheidene Opposition schleift sich die Wahrheit reiner; nur die Persönlichkeit, in der Larve dieser, bleibt tödtlich und häßlich. – Der Oekonon schreibt nicht immer für eine undankbare Menge. Wird diese Art Schriftstellerei auch oft mit armen Geistesprodukten verwechselt, und selten mit mehr, als mit einer mitleidigen, spöttischen Duldung belohnt: so bleiben doch geistige, humane Ideen stets Bürgen derer, die da geistiger kommen werden, Vorwärts, wie die Natur, ist das Panier des Denkers und des rationellen Oekonomen. –

Das Gebiet der Landwirthschaft aufzuklären, ist die humanste Arbeit der Zeit, zu der die Bessern alle freundlich geladen sind. Wie sich heute die Welt gestaltet, müssen Tausende gebildeter Menschen zum Pflug schwören, oder hungern, oder den Wanderstab nach den Einöden Amerikas und Rußlands nehmen, wo sie doch wieder nur den Pflug als Aegide des Lebens finden. Wie einst der Perfer die Sonne als feine nächste Wohlthäterin anbetete, so müssen bald in Europa Tausende aus andern zu voll gepfropften Ständen den Pflug verehren. In Deutschland sieht der Familienvater es bereits als eine gute Versorgung seines Sohnes an; wenn er als gemeiner Soldat, ein sonst so geflohener Zwang dient, untergebracht ist, und was sucht der traurige Auswanderer anders, als ein Bischen Erde, die ihm in seinem Vaterlande versagt wird? Die deutsche Erde wird zu enge, weil wir sie nur mit der rohen Kraft des gemeinen Ackermanns bauen; für rationelle Landwirthe hält sie noch für Hundert Millionen Menschen Raum.

3.

Written addendum to the transactions of sheep breeders - meeting in Brno, May 10, 1836.
By J.M. Freiherr von Ehrenfels.

The debate on sheep farming in No. 38 of this year's communications, in particular stimulated by Professor Nestler's contribution about the hereditary ability of noble progenitors has received great interest in public. It proves that the art of sheep breeding is struggling for principles and fundamentals and for this purpose urgently needs the auxiliary science, natural science, anatomy and physiology helping us to decipher an unknown truth from a known one. – I was so moved by the statements of Baron von Bartenstein (as not only the most excellent but at the same time the oldest promoter of Moravian sheep breeding), by the hints of Prof. Nestler (who emphasized the urgency of constancy as the basis of the pedigree formation so appropriately) and last but not least by the illuminations of Mr. Kunitz (who is personally unknown to me) about noble sheep breeding, that I allow myself in absentia to draw a few conclusions from these scattered statements and also to suggest further consequences.

I extoll in this debate the excellent observation of Professor Nestler, who with a deep look into the matter, in short words, indicated on p. 307: that he distinguishes genetic inheritance in relation to race from genetic inheritance in relation to breeding refinement. – The distinction is so scientifically fine that it might seem paradox to the crowd and yet it lies so true and deep in nature.

Just as the terms of refinement and breed formation are far apart themselves, so they are even more distant in the practical test of experience. The German merino sheep e.g. is refined but not a pedigree animal. The Hungarian Racka sheep is a pedigree sheep, but not a noble sheep. It is therefore up to us first to define: what pedigree of pure breeding and what refinement are.

Pedigree is, according to my school, a species of genus with individual properties and constant inheritance of the same. Refinement, on the other hand, is a variety of species brought about by genetic power, with one-sided properties without the power of heredity transmission.

According to this principle of natural science, a pedigree sheep would be a species of the genus sheep. But a species with special properties (e.g. fine wool, abundance of wool, ability to fatten, etc.) which individual property, organically impregnated by all parts of the body, forms its own type, which is faithfully inherited with the overall organization and is constantly passed on from two equally organized sexes to the son and daughter.

–So for example the Negretti sheep is a purebred animal, because it is so firmly constituted not only in its skin formation, from which its type of wool results, but also in all its organs, which keep this skin formation unchanged as a product of the overall organization, that it is transmitted organically, i.e. racially hereditary to distant descendants.
So, paired among themselves, the Hungarian Racka sheep with their individual characteristics continue to multiply for thousands of years, like the stag in the forest and, as a result of its firmly typed organization, produces wool and horn with a peculiar constant, like the stag its antlers and skin.

A refined sheep, on the other hand, would be a variety of the species produced by genetic force; a variety in which the genetic force has not permeated the whole organization, but only individual parts of the body, e.g. the skin. The skin, a product of the overall organization, is therefore neither independent nor constantly passed on. Hence are e.g. the German Merinos, and even the best part of them, coming from a mixture of the Negretti with the Electoral sheep, or vice versa, grafted or refined sheep.

They have altered their skin formation, which is an organic part of the body, through genetic manipulation of the overall organization. They yield finer wool than pure Negretti and give more wool than ultra-fine Elektorals.

Nevertheless, they have not been able to synthesize the whole structure of both species organically. The modified organization modifies the skin and the skin the wool. The non-consolidated type fails constant heredity transmission and so these so-called German Merinos, like all hybrid species, can scarcely be preserved with help of one or the other originals in their skin formation and the resulting wool type; in the next generation they are no longer formed in the same way as their parents. – -

Experience teaches the practical truth of this definition of pedigree and refinement, the scientific purity is based on Georges Cuvier, the greatest naturalist of our time. In his magnificent natural history rooms, he substantiates every word; from every single bone of prehistoric times he deciphers not only the genus, but also the species and variety of living and extinct animals. Cuvier proves in his comparative anatomy that sheep which have one
and the same skin structure and consequently one and the same wool, also present one and the same skeletal structure. And that therefore the constant inheritance of the wool is not only dependent on the skin, but on the contrary the skin itself remains dependent on the aggregate of the overall organization of all fluid and fleshy parts of the body.

The second thing I take the liberty of discussing from said debate is Mr. Kunitz's statement, page 306, second column. Mr. Kunitz freely states: "In noble sheep breeding it is essential to always have an ideal in mind. The supreme is the ideal and represents the noblest. But those who only strive for wool fineness are breeding one-sidedly. The whole organism in all its fine parts is to be considered." I add: not the formation of the skin alone, the overall organization, of which the skin itself is the product, had to be made the goal of pedigree formation, of constant heredity transmission. Because the individual parts form the whole unit, from the whole unit the modified skin formation results, from the skin formation the desired wool product follows and finally from the clearly defined type of the whole sheep body the constant inheritance of the breed with its productions results. Anyone who has won this view out of conviction has also gained the path to science as a breeder, who has understood nature in its creation.

In recent times we find clear evidence that an ideal with animal vitality and organic life can be realised as surely as Antonio Canova was carving a Venus out of a block of marble. Robert Bakewell idealized the English meat sheep. And how successful he was in holding on to this ideal, even in constant reproduction, is sufficiently expressed by the actual existence of the English meat sheep.

Now applying this reasoning, the next question in his sheep breeding would be: what can we accept as an ideal and as scientific naturalists, as an attainable ideal?

Already earlier and at another place I have expressed myself: what could be the ideal - model picture - for a noble sheep, namely a sheep that is as balanced as possible in fineness (according to Dollond 4-5 grade wool) and in the totality of a herd, and in conflict of all sexes and ages, wearing 2 pounds of pure washed wool on body. At the same time, however, it heredity transmits its overall organization constantly to its descendants. – That this ideal is achievable, is proven to me by so many realities of individual and in big herds living noble sheep, both bred by me and in several places in Germany. In the first transports of original Spanish sheep there were quite a number of such individuals, regardless of the statements and traditions that are critically mutilated today.

Thaler and I and historically older traditions called this noble sheep Eskurialschaf, which Mr. Peter fought passionately. In another place, when I have time, I shall actually elaborate on it and justify the name and the cause. - The name does not decide everything, but a lot. One has e.g. suggested instead of Eskurial, to use the name poor- and many-wooled electoral sheep, without considering, that these are no longer one and the same species, and thereby we endanger the category and race of the electoral sheep itself, by letting such fall into varieties. As soon as the Arabian bred in England had assumed the type of the English horse, it was correctly called from a natural history point of view, no longer Arabian; one did not divide the resulting horses into large and small Arabians, but called them "English

horse”. Both already had modified organizations as a whole, distinct bone and muscle formations in detail, and were no longer the same species genus horse. –

But stepping back from this occasional digression, a second difficult question arises from recent literature and experience:

"How should and can the ideal of the designated noble sheep be created through genetic power, represented in herds and made constantly hereditary?"

This principal question still forms the unsolved knot of sheep breeding science today. Fill for the discussion Anno 1837 the gap, which the deserved Baron Emanuel Bartenstein (president of the Brno Sheep Breeders Association) perceived in the years 1835 and 1836 in theoretical relation and give under the knowledge, enlightenment and growth, the debates of the European-famous Moravian-Silesian Sheep Breeders' Association a worthy subject of discussion, the breeding science a satisfactory answer.

We all, who have worked in this field of knowledge and experience with German perseverance and without presumption, with German thoroughness and who have conquered the golden fleece as German property, are growing old and perhaps would be irreplaceable if only one experience, one principle go down with us! All that is left of us here below is the spirit of our works: therefore perfection – perfection! In the smallest rests permanent greatness!!

Schriftlicher Nachtrag zu den Verhandlungen der Schafzüchter - Versammlung in Brünn, am 10. Mai 1836.
Von J. M. Freiherrn von Ehrenfels.

Die Debatte über Schafzucht in Nro. 38 der Mittheilungen v. J. zur Publizität gekommen, und vom Herrn Professor Nestler, über die Vererbungsfähigkeit edler Stammthiere, angeregt, hat ihrer Tendenz nach, das größte Interesse. Sie beweiset, daß die höhere Schafzucht nach Prinzipien und Grundsätzen ringt und hierzu die Hülfswissenschaft, Naturlehre, Anatomie und Physiologie, welche aus bekannten eine unbekannte Wahrheit entziffern helfen, dringend bedarf. –Ueberhaupt haben mich die Aeußerungen des Hrn.Baron v. Bartenstein, als des vorzüglichsten und zugleich ältesten Beförderers mährischer Schafzucht, die Andeutungen des Hrn. Prof. Nestler, der die Dringlichkeit der Konstanz als Basis der Rassebildung so entsprechend herausstellt, nicht minder die Lichtpunkte des mir persönlich unbekannten Hrn. Kunitz über edle Schafzucht, so bewegend angesprochen, daß ich mir abwesend erlaube, aus den zerstreuten Äußerungen nur ein Paar Resultate ziehend, Weiteres darauf vorzuschlagen.

Ich hebe aus der Debatte vorzüglich auswas Hr. Professor Nestler, mit tiefem Blick in die Sache, in kurzen Worten, S. 307 angedeudet: daß er die Vererbung in Bezug auf Rasse von jener in Bezug auf Veredlungunterscheide. – Die Distinktion ist so wissenschaftlich fein, daß sie der Menge paradox erscheinen dürfte und doch liegt sie so wahr und tief in der Natur.

So wie Veredlung und Rassebildung im Begriffe selbst weit von einander abstehen, so entfernen sie sich noch mehr in der praktischen Prüfungsschule Erfahrung. Das deutsche, Merinoschaf z. B. ist veredelt aber kein Rassethier. Das ungarische Zackelschaf ist Rasse-, aber kein Edelschaf Es beliebe uns daher vorerst zu definieren: was Rasse, was Veredlung seyen.

Rasse ist, nach meiner Schule, eine Species vom Genus mit individuellen Eigenschaften und konstanter Vererbung derselben. Veredlung dasgegen ist eine Varietät der Species durch genetische Kraft, mit einseitigen Eigenschaften ohne Vererbungsfähigkeit hervorgebracht.

Nach diesem Princip der Naturlehre wäre ein Rasseschaf eine Species vom Genus-Schaf; aber eine Species mit ausgezeichneter Eigenschaft (z. B. Feinwolligkeit, Wollreichthum, Mastungsfähigkeit u. f. w.) welche individuelle Eigenschaft, organisch durch alle Körpertheile imprägniert, einen eigenen Typum bildet, der sich mit der Gesammtorganisation getreu vererbt und zwischen zwei gleichorganisierten Geschlechtern konstant auf Sohn und Tochter übergehet.

– So z. B. ist das Negrettischaf ein Rassethier, weil es sich nicht nur in seiner Hautbildung, woraus dessen Wollart resultiert, sondern in all seinen Organen, welche diese Hautbildung, als Produkt der Gesammtorganisation unveränderterhalten, so fest konstituiert, daß es organisch d. i.rasseförmig auf ferne Nachkommen erblich übergehet. So pflanzt sich, unter sich gepaart, das ungarische
Zackelschaf mit seinen individuellen Eigenschaften Jahrtausende fort, wie der Hirsch im Walde und produziert in Folge seiner fest typirten Organisation Wolle und Horn, wie der Hirsch sein Geweih und seine Haut eigentümlich konstant.

Ein veredeltes Schaf dagegen wäre einedurch genetische Kraft hervorgegangene Varietätder Species; eine Varietät, bei der die genetische Kraft nicht die ganze Organisation, sondern nur einzelne Körpertheile durchdrungen hat, wie z. B.die Haut. Die Haut, ein Produkt der Gesammtorganisation, ist daher nicht selbstständig, noch weniger konstant vererblich. So sind z. B. die deutschen Merinos, und selbst der beste Theil derselben, aus einer Mischung des Negretti, mit dem Elektoralschaf erstanden, oder umgekehrt, veredelte Schafe.

Sie haben ihre Hautbildung das ist einen organischen Körperteil durch genetische Einwirkung auf die Gesamtorganisation verändert, sie geben feinere Wolle als pure Negretti und geben mehr Wolle als hochfeine Elektorals; sie haben aber nicht das ganze Gebilde beider Species organisch in sich vereinigen können, die modifizierte

Organisation modifiziert die Haut, die Haut die Wolle,der nicht konsolidierte Typus versagt die konstante Vererbung und so können diese sogenannten deutschen Merinos, wie alle Bastardarten, nur mit Nachhülfe ein oder anderen Originals, kaum in ihrer Hautbildung und der dadurch bezielten Wollart erhalten werden; sie sind in nächster Generation schon nicht mehr den Eltern gleich geformt. – -

Das praktisch Wahre dieser Definition von Rasse und Veredlung lehrt die Erfahrung, das wissenschaftlich Reine basiert auf Georges Cuvier, den größten Naturforscher unserer Zeit. In seinen naturhistorischen Prachtsälen belegt er jedes Wort; aus jedem einzelnen Knochen der Vorzeit entziffert er nicht nur das Genus, sogar die Species und Varietät der lebenden und ausgestorbenen Tiere. Cuvier beweist in seiner vergleichenden Anatomie, daß Schafe, welche ein und dieselbe Hautbildung und in Folge dieser ein und dieselbe Wolle trugen, auch ein und dasselbe Knochengebäude präsentieren, und daß daher die konstante Vererbung der Wolle nicht allein von der Haut, im Gegenteil die Haut selbst von dem Aggregat der Gesamtorganisation aller flüßigen und fleischigen Körpertheile abhängig bleibe.

Das Zweite, was ich mir aus besagter Debatte zu besprechen gestatte, ist die Aeußerung des Herrn Kunitz , Seite 306 zweite Spalte. Herr Kunitz sagt dem Sinne nach: »Bei edler Schafzucht sey es unerläßlich sich stets ein Ideal vor schweben zu lassen. Das höchstfeine sey Ideal und repräsentiere das Edelste. Doch wer blos nach Wollfeinheit strebet, züchtet einseitig. Der ganze Organismus in allen feinen Theilen sey zu berücksichtigen.« Ich setze bei: nicht die Hautbildung allein, die Gesammtorganisation, von welcher die Haut selbst das Produkt, mußte zum Zielpunkt der Rassebildung, der konstanten Vererbung gemacht werden, weil aus den einzelnen Theilen das Ganze, aus dem Ganzen die modifizierte Hautbildung, von der Hautbildung das gesuchte Wollprodukt undendlich aus dem fest ausgeprägten Typus des ganzen Schafkörpers die konstante Vererbung der Rasse mit ihren Produktionen resultiert. Wer diese Ansicht aus Überzeugung gewonnen hat, hat auch als Züchter den Weg zur Wissenschaft errungen, die Natur in ihrer Schöpfung verstanden.

In jüngst vergangener Zeit finden wir einensprechenden Beleg selbst ein Ideal mit thierischer Vitalität und organischem Leben, so sicher zu realisieren, als Antonio Canova aus einem Marmorblock eine Venus zu meißeln. Robert Bakewell idealisierte sich das englische Fleischschaf und wie gelungen und selbst in konstanter Fortpflanzung ihm dieses Ideal festzuhalten gelungen, spricht die thatsächliche Existenz des englischen Fleischschafes genüglich aus.

Dieses Raisonnement nun angewendet, wäre in seiner Schafzucht die nächste Frage: was wir als Ideal und als wissenschaftliche Naturforscher, als erreichbares Ideal, annehmen können?

Schon früher und an einem andern Orte habeich mich ausgesprochen: was beim Edelschaf Ideal –Musterbild – seyn könne, nämlich: ein Schaf, das an Feinheit möglichst ausgeglichen, nach Dollond 4 – 5 grädige Wolle, und in der Totaltät einer Heerde, und im Konflikt aller Geschlechts- und Altersklassen, 2 Pfund auf dem Körper reingewaschene Wolle trägt; dabei aber rasseförmigkonsolidiert seine Gesammtorganisation konstant auf
Nachkommen vererbet. – Daß dieses Ideal erreichbar sey, überzeugen mich so viele Wirklichkeiteneinzelner und in Mehrzahl lebender Edelschafe,sowohl von mir als an mehreren Orten Deutschlands gezüchtet. Auch sind in den ersten Transporten original spanischer Schafe derlei Individuen, der heute kritisch verstümmelten Aussagen und Traditionen ungeachtet, in ziemlicher Anzahl vorhanden gewesen.

Thaler und ich und geschichtlich ältere Traditionen haben dieses Edelschaf Eskurialschaf genannt, was Hr. Petri leidenschaftlich bekämpfet.

An einem andern Orte werde ich bei Muße thatsächlich darauf antworten und Name und Sache rechtfertigen. – Der Name entscheidet nicht Alles, doch Viel. Man hat z. B. vorgeschlagen: statt Eskurial, den Namen Arm- und Vielwolliges-Elektoralschaf zu gebrauchen, ohne zu bedenken,
daß diese nicht mehr eine und dieselbe Species sind, und wir dadurch Kategorie und Rasse des Elektoralschafes selbst gefährden, indem wir solches in Varietäten zerfallen lassen. Sobald der in England gezüchtete Araber den Typus des

englischen Pferdes angenommen hatte, nannte man's naturhistorisch richtig, nicht mehr Araber; theilte die daraus entfallenen Pferde nicht in große und kleineAraber, man nennt es das englische Pferd. Beide hatten schon modifizierte Organisationen im Ganzen, eigene Knochen- und Muskelbildung im Einzelnen, und waren nicht mehr dieselbe Species Genus-Pferd. –

Doch von dieser gelegentlichen Abschweifung zurück, bietet sich aus der neuesten Literatur und Erfahrung eine zweite schwierige Frage:

»Wie soll und kann man das Ideal des bezeichneten Edelschafes durch genetische Kraft firiren, in Herden darstellen und konstant vererblich machen?«

Diese Prinzipalfrage bildet heute noch den unaufgelösten Knoten der Schafzüchtungskunde. Erfülle für die Besprechung Anno 1837 die Lücke
aus, welche der verdiente Baron Bartenstein An. 1835 und 1836 in theoretischer Beziehung wahrgenommen und gebe unterm Wissen Aufklärung und Zuwachs, den Debatten des europäisch-berühmten mähr. schlef. Schafzüchter-Vereins einenwürdigen Besprechungs-Gegenstand, der Züchtungskunde eine realisierende Erledigung.

Wir Alle, die wir auf diesem Felde des Wissens und Erfahrens mit deutscher Beharrlichkeit und ohne Anmaßung auch mit deutscher Gründlichkeit gearbeitet und das goldene Vließ, als deutsches Eigenthum mit erobert haben, werden alt, und unersetzlich vielleicht wäre, wenn nur eine Erfahrung, ein Princip mit uns unterginge! Von uns bleibt hienieden nur übrig, der Geist unserer Werke: darum Vollendung – Vollendung! Im Kleinsten ruht dauernde Größe!!
